# MiR-151a: a robust endogenous control for normalizing small extracellular vesicle cargo in human cancer

**DOI:** 10.1186/s40364-023-00526-0

**Published:** 2023-10-20

**Authors:** Miranda Burdiel, Julia Jiménez, Carlos Rodríguez-Antolín, Álvaro García-Guede, Olga Pernía, Ana Sastre-Perona, Rocío Rosas-Alonso, Julián Colmenarejo, Carmen Rodríguez-Jiménez, María Dolores Diestro, Virginia Martínez-Marín, Oliver Higueras, Patricia Cruz, Itsaso Losantos-García, Héctor Peinado, Olga Vera, Javier de Castro, Inmaculada Ibáñez de Cáceres

**Affiliations:** 1 Biomarkers and Experimental Therapeutics in Cancer, IdiPAZ, Madrid, Spain; 2 Cancer Epigenetics Laboratory, INGEMM, La Paz University Hospital, Madrid, Spain; 3 Gynecologic Oncology Unit, La Paz University Hospital, Madrid, Spain; 4 Medical Oncology Department, La Paz University Hospital, Madrid, Spain; 5 Biostatistics Unit, La Paz University Hospital, IdiPAZ, Madrid, Spain; 6 Microenvironment and Metastasis Laboratory, Molecular Oncology Programme, Spanish National Cancer Research Center (CNIO), Madrid, Spain

**Keywords:** sEVs, miRNAs standardization, miRNA endogenous control in liquid biopsy

## Abstract

Small extracellular vesicles (sEVs) in the blood of cancer patients contain higher amounts of tumor markers than those identified as free-circulating. miRNAs have significant biomedical relevance due to their high stability and feasible detection. However, there is no reliable endogenous control available to measure sEVs-miRNA content, impairing the acquisition of standardized consistent measurements in cancer liquid biopsy. In this study, we identified three miRNAs from a panel of nine potential normalizers that emerged from a comprehensive analysis comparing the sEV-miRNA profile of six lung and ovarian human cancer cell lines in the absence of or under different conditions. Their relevance as normalizers was tested in 26 additional human cancer cell lines from nine different tumor types undergoing chemotherapy or radiotherapy treatment. The validation cohorts were comprised of 242 prospective plasma and ascitic fluid samples from three different human tumor types. Variability and normalization properties were tested in comparison to miR-16, the most used control to normalize free-circulating miRNAs in plasma. Our results indicate that miR-151a is consistently represented in small extracellular vesicles with minimal variability compared to miR-16, providing a novel normalizer to measure small extracellular vesicle miRNA content that will benefit liquid biopsy in cancer patients.

**Supplementary Information:**

The online version contains supplementary material available at 10.1186/s40364-023-00526-0.

To the Editor,

In the study of biological processes, the relevance of the results is determined by the normalization to an endogenous or reference control. In the cellular context, reference parameters have been widely studied and established [1, 2]. However, to date there is no endogenous control for microRNAs specific to the small extracellular vesicle (sEVs) compartment that provide a reproducible normalization. Given that sEV content has been found to be selective with respect to cellular content [3], an incorrect choice of endogenous control may bias the study results. The endogenous control most frequently used for sEV-miRNAs is miR-16 [4, 5], often without validating its homogeneity. Some studies have demonstrated the inadequacy of miR-16 as endogenous control in sEVs [6, 7], and rely on self-identified normalizers for their specific experimental context [8]. This scenario reveals the risk of disparity in the results published centered on sEVs-miRNAs as biomarkers and highlights the urgent need to identify a sEV-miRNA that can serve as an endogenous control.

Given that chemotherapy treatment represents external damage to the cell that modifies the gene/miRNA expression and methylation profiles [9–11], we considered this scenario ideal to identify stable sEVs-miRNAs candidates in these extreme situations. We first corroborated the isolation of 100 nm sEVs from the cell media and patients plasma using three alternative methodologies (Fig. S1). Next, we compared by small-RNAseq (Supplementary Methods) the sEVs-miRNA profiles obtained from cisplatin-sensitive and –resistant human cancer cell lines (Fig. S2) and found nine candidates with potential as reference controls (Table 1). miR-151a-3p, miR-22 and miR-221 showed a mean absolute internal variability (MIV) close to zero (|log2FC RvsS|≤ 0.3), higher average number of transcripts per kilobase million reads (TPM, ≥ 35) and lower variation (log2(Mean Expression) Variance ≤ 2) in 30 tumor types from the TCGA database (Table 1), strengthening their possible sEV content stability. miR-151a-3p showed the lowest MIV (|log2FC RvsS|= 0.084) compared to the other miRNAs and, importantly, it was 5.7 times less variable than miR-16 in sEVs from cell cultures, and 1.8 times in the TCGA tumors (0.32 vs. 0.58). This suggests that the differences in miRNA levels are much more evident in sEVs than in tumor tissue and would explain why to date miR-16 has been routinely used as endogenous control.

**Table 1:** List of the nine potential endogenous miRNAs identified by small RNA seq. In addition, the gold standard miR-16 normalizer and an external miRNA (miR-451) were used for normalizing purposes

MATURE-ID	Accession Number	Length (pb)	Mean Internal Variability	Mean Internal Variability in ovarian cancer samples	Mean Internal Variability in lung cancer samples	TPM per sample	Log2(Mean Expression) Variance in 30 cancer types (TCGA)
			**(|log2FC RvsS|)**	**(|log2FC RvsS|)**	**(|log2FC RvsS|)**		
**hsa-miR-151a-3p**	MI0000809	21	0.084	0.105	0.041	6759	0.32
**hsa-miR-22-5p**	MI0000078	22	0.169	0.066	0.376	39.33	0.58
**hsa-miR-502-3p**	MI0003186	22	0.193	0.263	0.052	39.5	0.61
**hsa-miR-221-3p**	MI0000298	23	0.257	0.356	0.054	851.67	1.67
**hsa-miR-1183**	MI0006276	27	0.276	0.35	0.129	112.5	NA
**hsa-miR-27a-3p**	MI0000085	21	0.278	0.296	0.242	747.67	6.67
**hsa-let-7i-5p**	MI0000434	22	0.297	0.441	0.011	9586.5	0.6
**hsa-miR-411-5p**	MI0003675	21	0.323	0.196	0.579	54.17	3.05
**hsa-miR-196b-5p**	MI0001150	22	0.329	0.218	0.55	87	3.67
**hsa-miR-16-5p**	MI0000070	22	0.477	0.347	0.737	103.34	0.58
**hsa-miR-451a**	MI0001729	22	3.014	3.37	2.304	8884.5	1.24

*R* Tumor cells resistant to platinum-based chemotherapy treatment, *S* Tumor cells sensitive to platinum-based chemotherapy treatment, *TPM* Transcripts per kilobase million, *TCGA* Tumor Cancer Genome Atlas

Using TaqMan-based qRT-PCR-specific amplifications for miR-151a-3p, miR-22, miR-221 and miR-16 (Supplementary methods) in sEVs from 32 human cell lines (Fig. S3A-D, Table S1), we found that miR-151a-3p had the lowest coefficient of variation amongst the miRNAs analyzed (CV = 0.061) and maintained its stability within the different tumor types (Fig. S3A), culture conditions (Fig. S4) and antitumor treatments such as cisplatin and carboplatin (Figs. S2, S3A). Of particular interest are our results regarding ionizing radiation (Fig. S3G, Table S2), where miR-151a-3p appeared to be the candidate with less cycle number variation (2.6 ± 0.055). To assess whether miRNA normalization ability was maintained for human plasma sEVs-miRNAs, we tested the levels of the four miRNAs in an exploratory cohort of 30 patient samples (Fig. S5A-D, Table S3) followed by analysis of miR-151a-3p and -16 levels in two validation cohorts of 172 and 70 samples (Fig. 1A-C, Figs. S6-S7, Tables S4-S6). miR-151a-3p showed the greatest stability and lower DM in human samples (Fig. 1D), including healthy volunteers (Fig. S6), different tumor types (Fig. 1E), cancer stages, sources (Fig. 1F) and post-treatment samples (Fig. S7). Moreover, normalizing miR-451a levels (Fig. 1C), a recently described sEV biomarker for the prediction of recurrence and prognosis in NSCLC patients [12], to miR-151a-3p and -16 showed partial correlation between them (R^2^_exploratory_ = 0.513; R^2^_validation_ = 0.732) (Fig. S5G, Fig. 1G). However, miR-151a distinguishes two patient groups from the controls based on values above the 75th or below the 25th percentiles, while this differentiation disappears with miR-16 (*p* = 0.0002878, Fig. 1H). Importantly, normalization against miR-151a, but not to miR-16, revealed differences in the sample origin (plasmatic or ascitic fluid, Fig. 1F), which could be beneficial for clinical use.

**Fig. 1:**
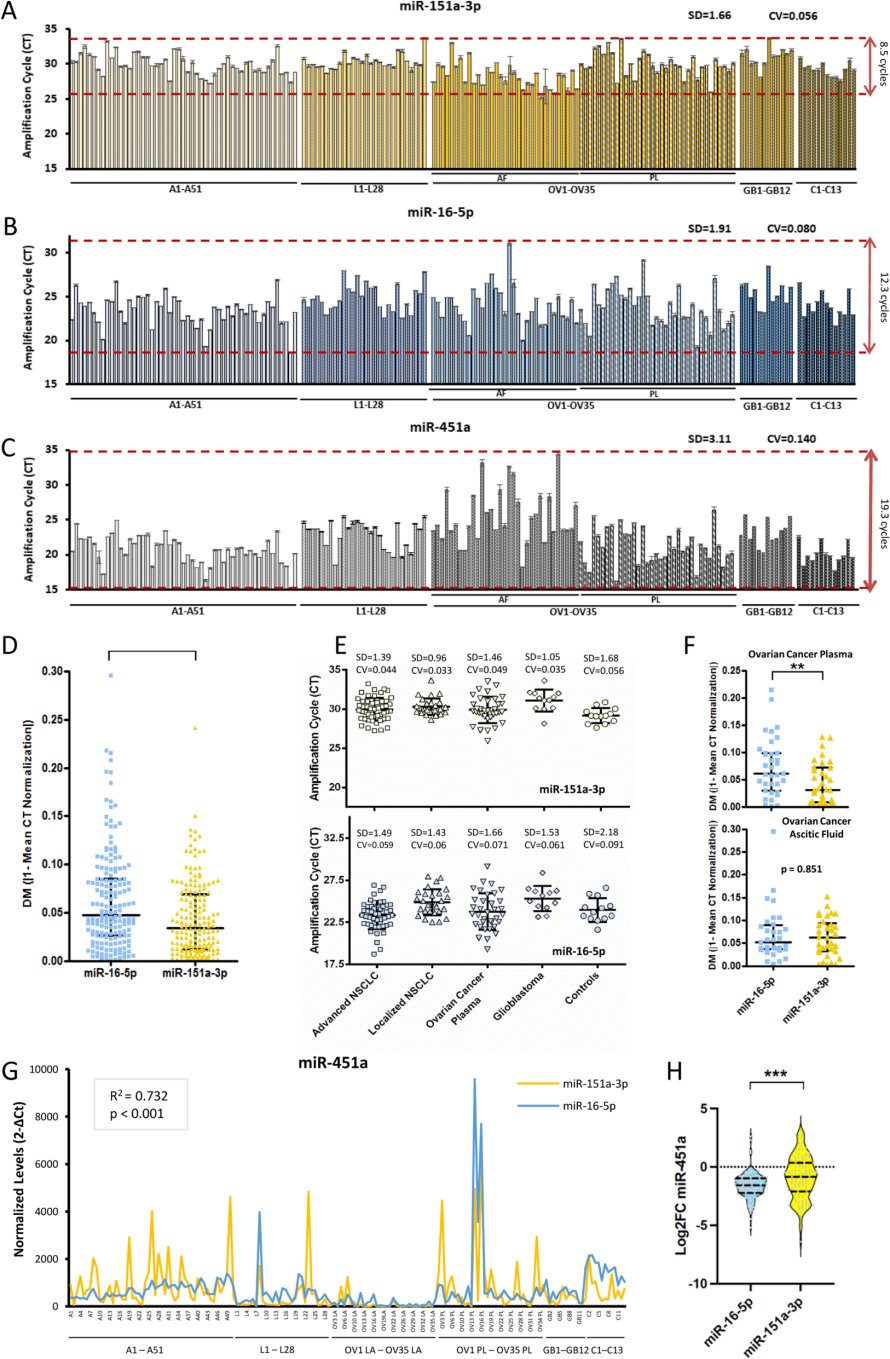
Amplification cycles of (**A**) endogenous sEV-miRNA-151a, **B** endogenous sEV-miRNA-16 and (**C**) miR-451a measured by qRT-PCR in 172 samples from blood and ascitic fluid from cancer patients and donors. We observed the lowest variation in cycle amplification when analyzing miR-151a compared to miR-16 within the different tumor types assessed and type of samples collected (SD: 1.66 cycles; CV: 0.056, versus SD: 1.91 cycles; CV: 0.080), finding nearly four fewer cycles of variation in miR-151a amplification (8.5 versus 12.3 cycles) (Fig. 1A, B). Compared to miR-451a amplification, which lacks normalizing features, these differences are three times higher (8.5 versus 19.3 cycles) (Fig. 1A and C). A1-A51 (51 advanced stage NSCLC), L1-L28 (28 early-stage NSCLC), OV1-35 (ovarian cancer patients; AF: Ascitic fluid; PL: Plasma), GB 1–12 (glioblastoma patients) and C1-13 (Healthy donors). **D** Distance to the mean (DM) was calculated using the normalized values in terms of absolute values. Mean CT normalization of each miRNA in each patient was calculated using the triplicate CT values ​​obtained by qRT-PCR from each sample, that was normalized against the mean value of all miRNA analyzed in the total samples evaluated in the assay. The distance to the mean of all miR-151a individual values obtained from each sample was statistically significant with respect to miR-16 (*p* < 0.001). **E** SD: Standard deviation of the mean value of the amplification cycle and CV: coefficient of variation (SD/mean) calculated related to the amplification cycle of miR-151a and miR-16 for each individual group of plasma samples. The coefficient of variation of the control samples is almost doubled in the case of miR-16 amplification (SD: 1.43 cycles; CV: 0.06 versus SD: 0.96 cycles; CV: 0.033). **F** SD, CV and DM values for plasma and ascetic fluid from ovarian cancer patients regarding miR-16 and 151a levels. miR-151a values remained closer to the overall mean (DM) than those of the miR-16 in the ovarian cancer plasma samples (*p* = 0.007) and, although this difference was not found when analyzing the ascitic fluid (*p* = 0.851). **G** miR-451 normalized levels with miR-151a or miR-16. A moderate correlation rate, primarily associated with those samples with the highest values of miR-451a, was maintained in this extended cohort when its levels were normalized by miR-151a or miR-16 (*R*.^*2*^ = 0.732), R: Correlation coefficient. Spearman’s nonparametric correlation test. **H** Violin plots illustrate the distribution of log2 fold change normalized miR-451a levels. The calibration against miR-151a would provide clear differentiation between two groups of patients behaving differently from the control value, with values above the 75th or below the 25th percentiles. Red dotted lines represent the median and quartiles. ***p < 0.01 and **p < 0.01

In summary, miR-151a stands out as the optimal endogenous control for normalizing sEVs-miRNA levels in both normal and tumor conditions, across a wide range of tumor types and antitumoral treatments. Its outstanding performance positions it as the most promising candidate thus far. Utilizing miR-151a in liquid biopsy tests will ensure reliable results for diverse basic, translational, and clinical studies.

### Supplementary Information

**Additional file 1: Figure S1.** Characterization of sEVs derived from human cancer cell lines, plasma samples and ascitic fluid. **Figure S2.** Cell viability after treatment with platinum-based drugs such as cisplatin (CDDP) and carboplatin (CBDCA). **Figure S3.** Amplification cycles of endogenous sEV-miRNAs in human cancer cell lines. **Figure S4.** Correlation between miR-151a-3p amplification cycle in sEVs isolated from the secretome of H1299, A2780, MCF7 and SW780 cell lines culture in the presence of either sEVs-depleted FBS (X axis) or absence of FBS (Y axis). **Figure S5.** Amplification cycles of endogenous sEV-miRNAs in 30 human samples. **Figure S6.** miR-151a-3p cycle amplification in the sEVs isolated from human samples. **Figure S7.** miR-151a-3p cycle amplification in the sEVs isolated from the second validation cohort of human samples. **Table S1.** Raw CT values of miR-151a-3p, miR-22-5p, miR-221-3p, miR-16-5p, miR-451a and their normalized levels using all the endogenous miRNAs in the sEVs compartment from human cancer cell lines and 293T. **Table S2.** Raw CT values of the endogenous miRNAs in sEVs compartment from 41M cells treated with radiotherapy at 0, 2, 4 and 6 Gy. **Table S3.** Raw CT values of miR-151a-3p, miR-22-5p, miR-221-3p, miR-16-5p, miR-451a and its normalized levels using all the endogenous miRNAs in circulating sEVs from 14 NSCLC patients (nine advanced and five early stage), six ovarian cancer patients (three plasma and three paired ascitic fluid), five glioblastoma patients and five healthy volunteers. **Table S4.** Raw CT values of miR-151a-3p, miR-22-5p, miR-221-3p, miR-16-5p, miR-451a and its normalized levels using all the endogenous miRNAs in circulating sEVs from 79 NSCLC patients (51 advanced and 28 early stage).** Table S5.** Raw CT values of miR-151a-3p, miR-22-5p, miR-221-3p, miR-16-5p, miR-451a and its normalized levels using all the endogenous miRNAs in circulating sEVs from 35 ovarian cancer patients. **Table S6.** Raw CT values of miR-151a-3p, miR-22-5p, miR-221-3p, miR-16-5p, miR-451a and its normalized levels using all the endogenous miRNAs in circulating sEVs from 12 glioblastoma patients and 13 healthy volunteers. **Table S7.** Cell line authentication using GenePrintR10 kit (Promega, USA). Genomics Service of the iiBm CSIC-UAM.

## Data Availability

The datasets generated and/or analyzed during the current study are available in the GEO repository, number GSE204944.

## Abbreviations

miRNAs: Micro-RNAs
sEVs: Small extracellular vesicles
TEM: Transmission electron microscopy
NTA: Nanoparticle tracking analysis
WB: Western blot
CDDP: Cisplatin
MIV: Mean Internal Variability
TCGA: The Cancer Genome Atlas
TPM: Transcripts per kilobase million reads
CBDCA: Carboplatin
SD: Standard deviation
CV: Coefficient of variation
DM: Distance to the mean
NSCLC: Non-small Cell Lung Cancer
