# Supplementary material for: MiR-151a: a robust endogenous control for normalizing small extracellular vesicle cargo in human cancer
Source: Biomark Res. 2023 Oct 20;11:94. doi: 10.1186/s40364-023-00526-0 (PMC10589979; doi:10.1186/s40364-023-00526-0)
Supplement: Supplementary file 1 — Additional file 1: Figure S1. Characterization of sEVs derived from human cancer cell lines, plasma samples and ascitic fluid. Figure S2. Cell viability after treatment with platinum-based drugs such as cisplatin (CDDP) and carboplatin (CBDCA). Figure S3. Amplification cycles of endogenous sEV-miRNAs in human cancer cell lines. Figure S4. Correlation between miR-151a-3p amplification cycle in sEVs isolated from the secretome of H1299, A2780, MCF7 and SW780 cell lines culture in the presence of either sEVs-depleted FBS (X axis) or absence of FBS (Y axis). Figure S5. Amplification cycles of endogenous sEV-miRNAs in 30 human samples. Figure S6. miR-151a-3p cycle amplification in the sEVs isolated from human samples. Figure S7. miR-151a-3p cycle amplification in the sEVs isolated from the second validation cohort of human samples. Table S1. Raw CT values of miR-151a-3p, miR-22-5p, miR-221-3p, miR-16-5p, miR-451a and their normalized levels using all the endogenous miRNAs in the sEVs compartment from human cancer cell lines and 293T. Table S2. Raw CT values of the endogenous miRNAs in sEVs compartment from 41M cells treated with radiotherapy at 0, 2, 4 and 6 Gy. Table S3. Raw CT values of miR-151a-3p, miR-22-5p, miR-221-3p, miR-16-5p, miR-451a and its normalized levels using all the endogenous miRNAs in circulating sEVs from 14 NSCLC patients (nine advanced and five early stage), six ovarian cancer patients (three plasma and three paired ascitic fluid), five glioblastoma patients and five healthy volunteers. Table S4. Raw CT values of miR-151a-3p, miR-22-5p, miR-221-3p, miR-16-5p, miR-451a and its normalized levels using all the endogenous miRNAs in circulating sEVs from 79 NSCLC patients (51 advanced and 28 early stage). Table S5. Raw CT values of miR-151a-3p, miR-22-5p, miR-221-3p, miR-16-5p, miR-451a and its normalized levels using all the endogenous miRNAs in circulating sEVs from 35 ovarian cancer patients. Table S6. Raw CT values of miR-151a-3p, miR-22-5p, miR-221- [file 40364_2023_526_MOESM1_ESM.docx]

**SUPPLEMENTARY information**

[**Supplementary Methods** 2](#_Toc145506133)

[**Cell culture** 2](#_Toc145506134)

[**Sample collection** 2](#_Toc145506135)

[**SEV characterization** 3](#_Toc145506136)

[*Transmission Electron Microscopy.* 3](#_Toc145506137)

[*Nanoparticle tracking analysis* 3](#_Toc145506138)

[*Western Blot* 3](#_Toc145506139)

[**RNA extraction and Small RNAseq (miRNAseq)** 4](#_Toc145506140)

[**qRT-PCR** 4](#_Toc145506141)

[**Statistical Analysis** 5](#_Toc145506142)

[**Supplementary Methods References** 5](#_Toc145506143)

[**Supplementary Figures and tables** 6](#_Toc145506144)

[**Figure S1.** 6](#_Toc145506145)

[**Figure S2.** 7](#_Toc145506146)

[**Figure S3.** 8](#_Toc145506147)

[**Figure S4** 9](#_Toc145506148)

[**Figure S5** 10](#_Toc145506149)

[**Figure S6** 12](#_Toc145506150)

[**Figure S7** 13](#_Toc145506151)

[**Table S1** 14](#_Toc145506152)

[**Table S2** 15](#_Toc145506153)

[**Table S3** 16](#_Toc145506154)

[**Tables S4** 17](#_Toc145506155)

[**Table S5** 19](#_Toc145506156)

[**Table S6** 20](#_Toc145506157)

[**Table S7.** 21](#_Toc145506158)

# **Supplementary Methods**

## **Cell culture**

A total of 31 human cell lines representing the most frequent human cancer types were used including lung (H23, H1299, H727, H1975 and A549), colon (HT-29 and LoVo), pancreas (IMIM-PC2 and PANC-1), breast (MCF7), liver (Hep EPG2), bladder (SW 780), head and neck (OSC-20, CAL27 and CAL-33), ovarian cancers (A2780 and OVCAR-3) and glioblastoma (T98G, LN-229, U-251 and U-87), as well as 293T cells. These cell lines were purchased from the ATCC (Manassas, VA, USA) or ECACC (Sigma-Aldrich, Spain) and authenticated by the Genomics Department of the Alberto Sols Biomedical Research Institute (iiBm) CSIC-UAM, through microsatellite analysis of specific loci within the Human Genome using the GenePrint 10 kit (Promega, USA) (Table S7). Cisplatin (CDDP) resistant variants H23R, OVCAR-3R and A2780R were established previously by our group (1-5). Carboplatin (CBDCA) resistant subtypes H23 and A2780 were established by exposing cells to increasing doses of carboplatin as described in the literature (4), obtaining a final CBDCA concentration of 2.5 and 1.25 µg/mL, respectively (Figure S2). The CDDP-sensitive and resistant ovarian cancer cell lines 41M and 41MR were kindly provided by Dr. Kelland (UK). FBS was depleted of bovine sEVs by ultracentrifugation at 100,000 × g for 16h at 4ºC. All cell lines were maintained in RPMI or DMEM supplemented with 10% sEV-depleted FBS, according to manufacturer specifications. For each experiment, the original sensitive and resistant subtypes were cultured for 72h in sEV-depleted FBS media before sEV isolation. Additionally, a panel of these cell lines was cultured in the absence of FBS to compare with sEV-depleted FBS. The IC50 (µg/mL) values comparing resistant versus sensitive phenotypes are also included in Supplementary Figure 1. The CDDP-sensitive 41M cell line was irradiated at doses of 2, 4 and 6 Gy using a Mark I-30 Cesium-137 irradiator (J.L. Shepherd and Associates, CA, USA). Cells were cultured for 72 h following irradiation, when the media was collected for sEV isolation. Individual assays were performed in duplicate. All cell lines under different experimental conditions were used to validate the normalizing properties of the miRNA candidates identified after the miRNome screening performed in the paired cisplatin-sensitive and resistant cell lines H23, A2780 and 41M along with the analysis of miR-16 levels.

## **Sample collection**

A total of 172 plasma samples were collected prospectively before receiving any antitumoral treatment from 51 advanced NSCLC patients (stages IIIA to IV), 28 patients at localized NSCLC stages (stages IA to IIIA), 12 glioblastoma patients (GB), 35 plasma and ascites fluid paired samples from ovarian cancer patients and plasma from 13 healthy donors, following the criteria of the medical oncology division of La Paz University Hospital. All samples were processed following the standard operating procedures with the appropriate approval of the Human Research Ethics Committees, including informed consent within the context of research (HULP: PI-3508 and -5063). All samples were processed within the first 30 minutes of collection using Vacutainer EDTA blood collection tubes in the case of blood and hemolyzed samples were discarded for the study as recommended (6).

## **SEV characterization**

Unbroken cells and debris were removed from the culture media by centrifugation at 7480 rpm for 5 min; for both the ascitic fluid and the blood plasma samples, two centrifugations at 5130 rpm and 14000 rpm for 10 min and 20 min respectively were performed. Samples were stored at –80C° until use. Cell line-derived sEVs used for small RNA-seq and for qRT-PCR validation, were isolated by miRCURY Exosome Cell Isolation Kit starting from 1 mL of sEV depleted FBS cell culture media (Qiagen, Germany). Circulating sEVs were obtained with the exoRNeasy Serum/Plasma Midi Kit (Qiagen, Germany). All samples followed a 0.22 µm filtration step that was included in the protocol directions. Transmission Electron Microscopy (TEM), Nanoparticle Tracking Analysis (NTA) and Western Blot (WB) were used for sEV characterization.

### *Transmission Electron Microscopy.*

The analysis of the size and morphology of the sEVs by Transmission Electron Microscopy was conducted in the TEM Department of the Autonomous Univesity of Madrid School of Medicine. SEVs from cisplatin-resistant cells H23R, A2780R and 41MR were fixed in 100 µl of 0.1 M paraformaldehyde (PFA) of 2% phosphate buffer (pH 7.4) and samples were then transferred onto Formvar/carbon-coated copper grids. The grids were subsequently fixed with 1% w/v glutaraldehyde in PBS and washed several times in distilled water. After the washes, the grids were contrasted with 2% uranyl oxalate and soaked in a mixture of 0.4% uranyl acetate and 1.8% methylcellulose. The samples were examined in a JEOL JEM-1010 transmission electron microscope (JEOL, USA), using a working voltage of 100 kV and a 120,000 magnification. Analysis of the samples was conducted using the DigitalMicrograph program for the visualization and processing of images.

### *Nanoparticle tracking analysis*

Nanoparticle tracking analysis (NTA) was performed using a NanoSight LM10 microscope equipped with NTA software v3.0 (Malvern, UK) in the cisplatin-resistant sEV derived cells H23, A2780 and 41M, and in a representation of plasma sEVs isolated from NSCLC, ovarian and glioblastoma patients. Background extraction was applied and the automatic setting for minimum expected particle size, minimum track length and blur settings were employed. Three 30 s recordings at 30 frames per second were taken for each sample, which were diluted at 1:400-1:100 depending on the sample concentration in sterile filtered PBS.

### *Western Blot*

20 µg of cisplatin-resistant H23R, A2780R and 41MR whole cell extracts and their sEV-rich pellets obtained from the cell media were resuspended in 1X RIPA buffer, separated on a polyacrylamide gel, and transferred to a polyvinylidene difluoride (PVDF) membrane. The membrane was blocked with bovine serum albumin and incubated first with TSG101 antibody (ab30871, Abcam, 1: 1000) and CD81 antibody (sc23962, Santa Cruz, 1:1000) and then with species-specific horseradish peroxidase-labelled secondary antibodies (Thermo Scientific, MA, USA). Proteins were detected by enhanced chemiluminescence (Thermo Scientific, MA, USA).

## **RNA extraction and Small RNAseq (miRNAseq)**

miRNA content from circulating sEVs was obtained from plasma and ascites fluid samples with an exoRNeasy Serum/Plasma Midi Kit (Qiagen, Germany). Cisplatin-sensitive/resistant paired H23S/H23R, A2780S/A2780R and 41S/41R cells (Supplementary Figure 1) were used for the Small-RNAseq. RNA was extracted using a miRCURY RNA Isolation Kit -Cell and Plant (Exiqon, Denmark) according to manufacturer instructions. RNA was quantified with a NanoDrop ND-1000 spectrophotometer (Thermo Fisher Scientific, USA) and with a Qubit 4 fluorometer (Invitrogen, Thermo Fisher Scientific, USA), then analyzed with Arraystar (Arraystar Inc., MD, USA) as follows: Total RNA of each sample was used to prepare the miRNA sequencing library; 3' and 5' adapter ligation, cDNA synthesis, PCR amplification and size selection between 130 and 150 bp (corresponding to 15‒35 nucleotides (nt) of miRNAs) were performed. The DNA was sequenced with 51 cycles using the Illumina HiSeq 2000 sequencer (Illumina Inc., CA, USA). The trimmed reads (length >= 15 nt and adapter removal) were aligned to human pre-miRNA in miRBase 21 (7) using NovoAlign software (8) and miRNA read counts were normalized as tag counts per million (TPM) alignments (7). Calculating the miRNA levels, reads with counts of less than 2 were discarded. Fold Change (log2FC) and p-values were computed for comparisons between samples (resistant versus sensitive phenotype). Those with the lowest Log2FC value with at least one count per million reads under all experimental conditions were then selected, and the hg19 version was used as a reference annotation. GEO repository number: GSE204944 (restricted access until article acceptance).

## **qRT-PCR**

The nonspecific retrotranscription of all miRNAs from each sample was conducted using the TaqMan TM Advanced miRNA cDNA Synthesis Kit (Thermo Fisher Scientific, USA), according to manufacturer instructions. Quantitative analysis of each specific miRNA was performed using the next TaqMan Advanced miRNA assay for each candidate: hsa-miR-151a-3p: 477919_mir; hsa-miR-22-5p: 477987_mir; hsa-miR-221-3p: 477981_mir; hsa-miR-16-5p: 477860_mir; and hsa-miR-451a: 478107_mir). TaqMan Universal PCR Master Mix (Thermo Fisher Scientific, USA) was used for qPCR amplification. All samples were analyzed in triplicate with a HT7900 Real-Time PCR System thermocycler (Applied Biosystems, USA) according to these settings: 10 min at 95ºC and 40 cycles of 15 s at 95ºC followed by 1 min at 60ºC. The analysis of the results was performed with RQ Manager software (Thermo Fisher Scientific, USA) and the relative quantification of miR-451a levels was based on the comparative method 2-ΔCt, calculating the value of ΔCt by subtracting endogenous Ct value to miR-451a Ct value. In the case of the radiotherapy treatment, the values from 41M mock cells were used as calibrator.

## **Statistical Analysis**

miRNA levels were represented by the mean values ± S.D. from at least three replicates. The coefficient of variation (CV) was calculated as the ratio of the standard deviation to the mean (CV=SD/mean). Variance analysis was performed using Levene test. Statistical analysis of the effect of radiotherapy in cycle number variation was performed using Student’s t test (paired; two-tailed) with a significance of p < 0.001 in all cases.

For a specific miRNA, triplicate Ct values ​​obtained by qRT-PCR from each sample were normalized against the mean value of the same miRNA analyzed in the total samples evaluated. These normalized values were used to calculate the distance from the mean (DM) in terms of absolute values, performing the Wilcoxon signed-rank test to analyze them. Correlation analysis between normalized levels of miR-451a using miR-151a or miR-16 as endogenous controls were carried out with a non-parametric Spearman test.

All statistical analyzes were performed with SAS 9.3 (SAS Institute, Cary, NC, USA) and RStudio (version 1.1.423). Values of p<0.05 were considered as statistically significant, being * p<0.05, ** p<0.01 and *** p<0.001.

# **Supplementary Methods References**

1. Vera O, Rodriguez-Antolin C, de Castro J, Karreth FA, Sellers TA, Ibanez de Caceres I. An epigenomic approach to identifying differential overlapping and cis-acting lncRNAs in cisplatin-resistant cancer cells. Epigenetics. 2018;13(3):251-63.

2. Vera-Puente O, Rodriguez-Antolin C, Salgado-Figueroa A, Michalska P, Pernia O, Reid BM, et al. MAFG is a potential therapeutic target to restore chemosensitivity in cisplatin-resistant cancer cells by increasing reactive oxygen species. Transl Res. 2018;200:1-17.

3. Pernia O, Sastre-Perona A, Rodriguez-Antolin C, Garcia-Guede A, Palomares-Bralo M, Rosas R, et al. A Novel Role for the Tumor Suppressor Gene ITF2 in Tumorigenesis and Chemotherapy Response. Cancers. 2020;12(4).

4. Vera O, Jimenez J, Pernia O, Rodriguez-Antolin C, Rodriguez C, Sanchez Cabo F, et al. DNA Methylation of miR-7 is a Mechanism Involved in Platinum Response through MAFG Overexpression in Cancer Cells. Theranostics. 2017;7(17):4118-34.

5. Soto JA, Rodriguez-Antolin C, Vera O, Pernia O, Esteban-Rodriguez I, Dolores Diestro M, et al. Transcriptional epigenetic regulation of Fkbp1/Pax9 genes is associated with impaired sensitivity to platinum treatment in ovarian cancer. Clinical epigenetics. 2021;13(1):167.

6. McDonald JS, Milosevic D, Reddi HV, Grebe SK, Algeciras-Schimnich A. Analysis of circulating microRNA: preanalytical and analytical challenges. Clin Chem. 2011;57(6):833-40.

7. Kozomara A, Griffiths-Jones S. miRBase: annotating high confidence microRNAs using deep sequencing data. Nucleic Acids Res. 2014;42(Database issue):D68-73.

8. Novocraft technologies. <http://www.novocraft.com/> [Available from: <http://www.novocraft.com/>.

# **Supplementary Figures and tables**


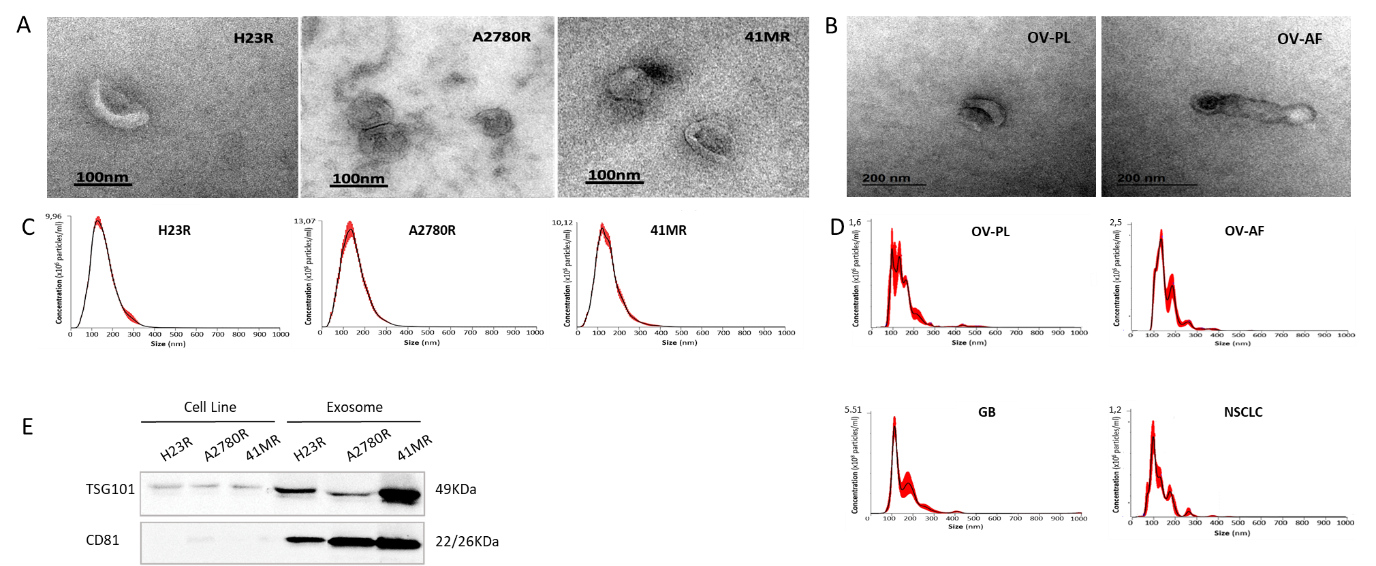


**Figure S1.** **Characterization of sEVs derived from human cancer cell lines, plasma samples and ascitic fluid.** Visualization of **(A)** H23R, A2780R and 41MR cell line-derived sEVs and **(B)** circulating sEVs obtained from plasma and ascitic fluid from an ovarian cancer patient by using transmission electron microscopy (TEM). Images were taken at 120,000 magnifications. TEM revealed the presence of approximately 100 nm cup or spherical shape structures in cell-derived sEVs as well as in plasma and ascites fluid. Particle tracking of sEVs from **(C)** the cell lines H460R, A2780R, 41MR; and from **(D)** plasma and ascitic fluid of three patients with NSCLC, glioblastoma and ovarian cancer using Nanosight (NTA). NTA also revealed that the size of the major particles was around 100 nm. **(E)** Western blot (WB) was used as a third method for sEV characterization using antibodies targeting two common sEV markers located in sEV membranes: tetraspanin molecules TSG101 and CD81. Both markers were enriched in sEVs isolated from the cisplatin resistant H23R, A2780R and 41MR cell cultures compared to whole cell extracts, confirming the successful sEV isolation.

**
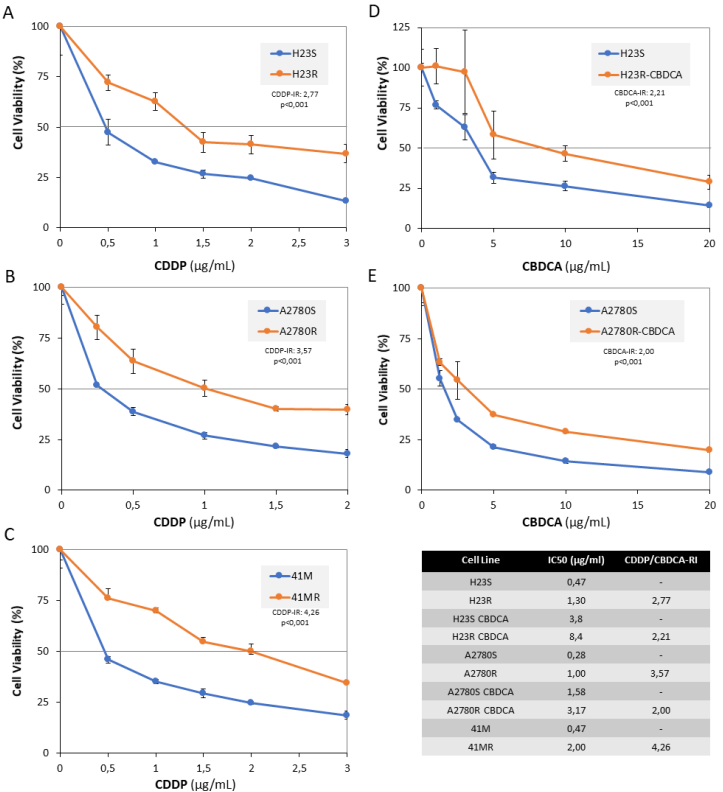
**

**Figure S2. Cell viability after treatment with platinum-based drugs such as cisplatin (CDDP) and carboplatin (CBDCA).** The curves show the resistance to CDDP of the (A) H23R, (B) A2780R, (C) 41MR cells; and to CBDCA from (D) H23R-CBDCA cells and (E) A2780R-CBDCA. Data was normalized compared to the untreated control. IC50 is the concentration that induces the death of 50% of the cell population. The Resistance Index (RI) was calculated as IC50 of the resistant cell line / IC50 of the sensitive cell line. p < 0.001 was considered as a significant change in drug sensitivity (Student’s t test).

**Figure S3.** **Amplification cycles of endogenous sEV-miRNAs in human cancer cell lines.** **(A-C)** Candidate endogenous miRNA controls quantified by qRT-PCR in the sEVs of the secretome of six CDDP paired sensitive and resistant cell lines, 19 additional cell lines from different tumor types and three additional cell lines treated with CBDCA or RT. SD: Standard deviation of the mean value of the amplification cycle, CV: coefficient of variation (SD/mean). The lowest variability among the different samples analyzed was found in miR-151a, with a standard deviation of 1.73 cycles and a coefficient of variation of 0.061, compared to candidates miR-22-5p (SD: 2.06; CV: 0.072) and miR-221-3p (SD: 2.17; CV: 0.093). **(D)** Levels of endogenous miR-16 quantified by qRT-PCR in the sEVs, in the same experimental groups as S3A-C. Higher variability in comparison with miR-151a and similar to miR-221 (SD: 2.44; CV: 0.10) was observed. **(E)** Mean CT normalization of each miRNA in each independent cell line. Triplicate CT values ​​obtained by qRT-PCR from each sample were normalized against the mean value of all miRNAs analyzed in the total samples evaluated in the assay. miR-151 values remained close to the overall mean obtained from all the cell lines analyzed. **(F)** Distance from the mean (DM) was calculated using the normalized values in terms of absolute values and Wilcoxon signed-rank test was used for the analysis. DM was statistically significant when compared to miR-16 and -221 (p<0.05) while no significant differences were observed with miR-22 (p=0.125). The variability observed in the DM was not statistically different between miR-16 and miR-221 (p=0.765). **(G)** Cycle number variation for each of the four miRNAs analyzed in the 41M cells treated with radiotherapy at 2, 4 and 6 Gy using 41M Mock cells without radiotherapy treatment as calibrator. Number of cycles observed for miR-151a amplification varied 2.7 times less compared to miR-16 (2.6 + 0.055 versus 7.0 + 0.075; p<0.001); 2.5 times less compared to miR-221 (2.6 + 0.055 versus 6.5 + 0.054; p<0.001) and 1.99 times less compared miR-22 (2.6 + 0.055 versus 5.2 + 0.63; p<0.05). The cycle variation in the case of miR-221 and -22 was also significantly lower than miR-16 for the three radiotherapy doses tested (5.21 +0.63 and 6.47 +0.05 versus 7.0 +0.075 respectively; p<0.05). ***p<0.001 and **p<0.01 (compared to miR-16) and **###** p<0.001 and **##**p<0.01 (compared to miR-151a-3p) **(H)** Relative miR-451a levels normalized with miR-151a-3p, miR-16-5p, miR-221-5p and miR22-5p in sEVs from human cancer cell lines. The normalization of miR-451a levels relative to the candidate miR-151a and the miR-16 standard used showed a significant correlation (p<0.001), with an intermediate correlation rate of 0.685. Normalization to miR-22 and miR-221 showed as expected a slightly better correlation rate with miR-151a normalization (R=0.798 and 0.741 respectively). R: Correlation coefficient. Spearman’s nonparametric correlation test.

**Figure S4:** Correlation between miR-151a-3p amplification cycle in sEVs isolated from the secretome of H1299, A2780, MCF7 and SW780 cell lines culture in the presence of either sEVs-depleted FBS (X axis) or absence of FBS (Y axis). Samples were quantified by qRT-PCR in a HT7900 Real Time PCR System thermocycler (Applied Biosystems, USA). R^2^ shows the correlation between the two types of samples. We did not observe differences in the levels of miR-151 between sEVs-depleted FBS and the absence of FBS, showing a positive correlation between both conditions in the same cell line (R^2^= 0.08718).

**Figure S5. Amplification cycles of endogenous sEV-miRNAs in 30 human samples.** **(A-C)** Candidates endogenous miRNA controls and **(D)** endogenous miR-16, all quantified by qRT-PCR in the plasma sEVs of 14 NSCLC patients (nine advanced and five early stage), six ovarian cancer patients (three plasma and three paired ascitic fluid), five glioblastoma patients and five healthy volunteers. miR-151a is the candidate with the lowest variability and coefficient of variation within the different samples analyzed (SD: 1.16 cycles; CV: 0.062), followed by miR-22 (SD: 1.89 cycles; CV: 0.075), miR-221 (SD: 2.17; CV: 0.077) and finally miR-16 (SD: 2.44: CV: 0.076). These differences were statistically significant when comparing miR-151a with miR-16 (p=0.02; Levene test). SD: Standard deviation, CV: coefficient of variation (SD/mean). **(E)** Mean CT normalization of each miRNA in each patient. The mean CT normalization of each miRNA in every independent sample tested revealed that miR-151a values remained the closest to the overall mean obtained from all the patients tested. For a specific miRNA, triplicate CT values ​​obtained by qRT-PCR from each sample were normalized against the mean value of the same miRNA analyzed in the total samples evaluated. **(F)** Distance from the mean (DM) was calculated using the normalized values in terms of absolute values and Wilcoxon signed-rank test was used for the analysis. We observed statistically significant DM values different from all miRNAs tested of p=0.001 compared to miR-16, p<0.005 to miR-221 and p<0.05 to miR-22. **(G)** Relative miR-451a levels normalized with miR-151a-3p, miR-16-5p, miR-221-5p and miR22-5p in sEVs from human plasma and ascitic fluid samples. We found a very weak correlation rate of R=0.513 (Spearman correlation) when the levels of miR-451a were normalized to miR-151a or miR-16. The right axis is compared only to the normalization of the miR-451a with the miR-22-5p (brown line).R: Correlation coefficient. Spearman’s nonparametric correlation test. ***p<0.001, **p<0.01 and *p<0.05.


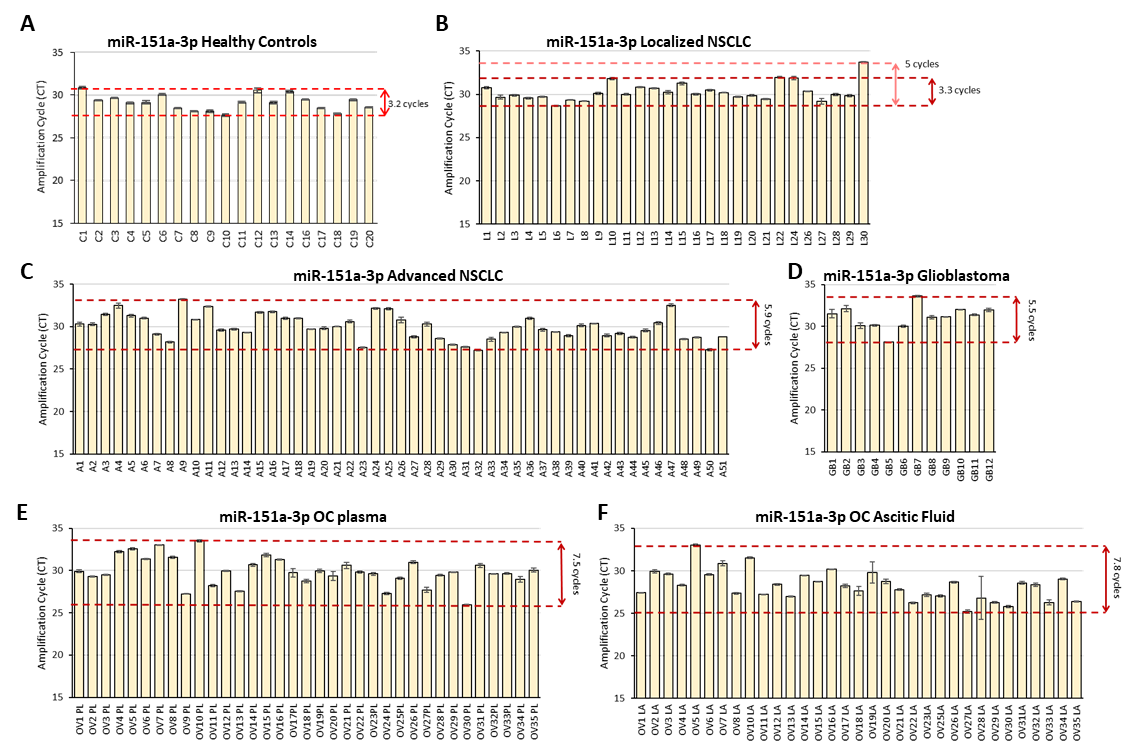


**Figure S6. miR-151a-3p cycle amplification in the sEVs isolated from human samples. (A)** Plasma from 19 healthy volunteers. **(B)** Plasma from 28 Non-small cell lung cancer patients at early stage. **(C)** Plasma from 51 Non-small cell lung cancer patients at advanced stage. The global variability of 8.5 cycles for miR-151a found in Figure 4 drops to 3.2 and 3.3 cycles in the case of healthy individuals (S6A) and patients with localized stages NSCLC (S6B), and to 5.9 cycles in the case of patients with advanced stages NSCLC (S6C). **(D)** Plasma from 12 glioblastoma patients. **(E)** Plasma from 35 ovarian cancer patients. **(F)** Ascitic fluid from 35 ovarian cancer patients. In patients with glioblastoma, a slightly lower variability is identified (5.5 cycles) (S6D) and finally the greatest variability is observed in patients with ovarian cancer with variations of 7.5 and 7.8 depending on the plasma or ascitic fluid origin (S6F).


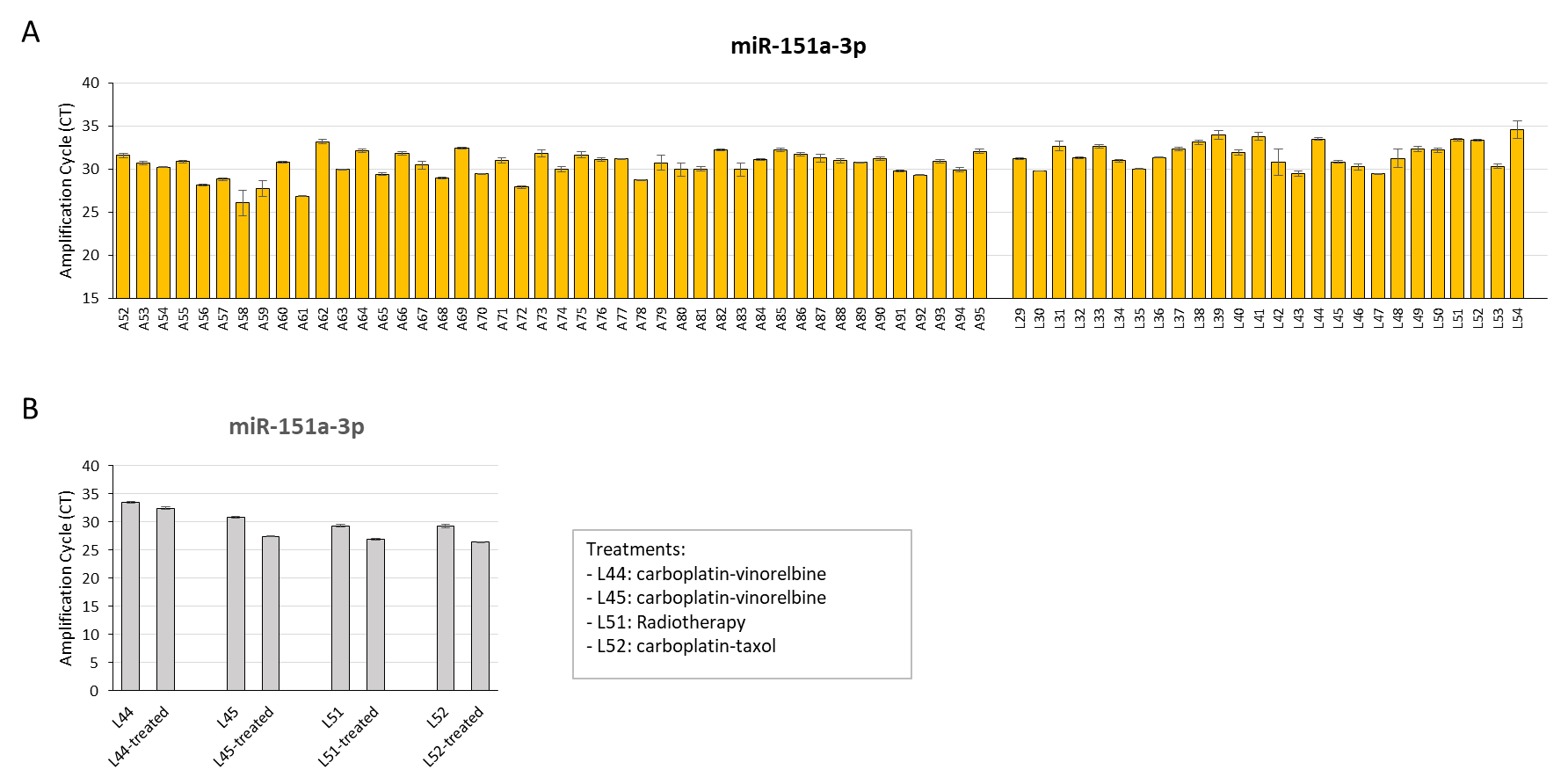


**Figure S7. miR-151a-3p cycle amplification in the sEVs isolated from the second validation cohort of human samples.** (A) 44 additional plasma samples from advanced NSCLC (A52-A95) and 26 localized NSCLC (L29-L56), and (B) 4 localized NSCLC patients before and after different chemotherapy and radiotherapy treatments.

**Table S1. Raw CT values of miR-151a-3p, miR-22-5p, miR-221-3p, miR-16-5p, miR-451a and their normalized levels using all the endogenous miRNAs in the sEVs compartment from human cancer cell lines and 293T.** Normalized CT values are compared to the triplicate CT values obtained by qRT-PCR from each sample normalized against the mean value of all miRNA analyzed in the total samples evaluated in the assay. Distance from the mean (DM) was calculated using the normalized values in terms of absolute values |1-Normalized CT values|.

|  | **CT values** | | | | | **Normalized miR-451a values** | | | | **Normalized CT values** | | | | **DM** | | | |
| --- | --- | --- | --- | --- | --- | --- | --- | --- | --- | --- | --- | --- | --- | --- | --- | --- | --- |
|  | **miR-151a-3p** | **miR-22-5p** | **miR-221-3p** | **miR-16-5p** | **miR-451a** | **miR-151a-3p** | **miR-22-5p** | **miR-221-3p** | **miR-16-5p** | **miR 151a-3p** | **miR 22-5p** | **miR 221-3p** | **miR 16-5p** | **miR 151a-3p** | **miR 22-5p** | **miR 221-3p** | **miR 16-5p** |
| **H1299** | 26.499 | 27.932 | 22.339 | 23.436 | 23.727 | 6.852 | 18.467 | 0.383 | 8.205 | 0.940 | 0.971 | 0.961 | 0.978 | 0.0598 | 0.0289 | 0.0390 | 0.0221 |
| **H727** | 27.672 | 30.586 | 22.910 | 25.865 | 27.290 | 1.307 | 9.843 | 0.048 | 3.729 | 0.982 | 1.063 | 0.986 | 1.079 | 0.0182 | 0.0634 | 0.0144 | 0.0792 |
| **H1975** | 27.221 | 26.238 | 21.896 | 24.480 | 28.199 | 0.508 | 0.257 | 0.013 | 0.761 | 0.966 | 0.912 | 0.942 | 1.021 | 0.0342 | 0.0878 | 0.0580 | 0.0215 |
| **A549** | 28.943 | 28.920 | 24.759 | 27.732 | 31.265 | 0.200 | 0.198 | 0.011 | 0.868 | 1.027 | 1.005 | 1.065 | 1.157 | 0.0269 | 0.0055 | 0.0651 | 0.1571 |
| **HT29** | 27.443 | 30.973 | 22.734 | 23.678 | 29.437 | 0.252 | 2.925 | 0.010 | 0.185 | 0.974 | 1.077 | 0.978 | 0.988 | 0.0263 | 0.0769 | 0.0220 | 0.0120 |
| **LoVo** | 27.902 | 26.966 | 20.536 | 22.136 | 28.321 | 0.762 | 0.391 | 0.005 | 0.138 | 0.990 | 0.938 | 0.883 | 0.924 | 0.0101 | 0.0625 | 0.1165 | 0.0764 |
| **IMIM PC2** | 29.742 | 29.597 | 22.399 | 25.739 | 30.726 | 0.506 | 0.460 | 0.003 | 0.315 | 1.055 | 1.029 | 0.964 | 1.074 | 0.0552 | 0.0290 | 0.0364 | 0.0740 |
| **PANC1** | 27.183 | 28.098 | 22.237 | 22.437 | 28.327 | 0.453 | 0.857 | 0.015 | 0.169 | 0.964 | 0.977 | 0.957 | 0.936 | 0.0356 | 0.0231 | 0.0434 | 0.0638 |
| **MCF7** | 26.763 | 29.295 | 25.124 | 21.316 | 28.749 | 0.253 | 1.462 | 0.081 | 0.058 | 0.950 | 1.019 | 1.081 | 0.889 | 0.0505 | 0.0185 | 0.0808 | 0.1106 |
| **T98G** | 27.507 | 26.845 | 22.736 | 21.533 | 27.128 | 1.306 | 0.825 | 0.048 | 0.208 | 0.976 | 0.933 | 0.978 | 0.898 | 0.0241 | 0.0667 | 0.0219 | 0.1015 |
| **LN229** | 29.033 | 29.426 | 22.970 | 26.723 | 30.612 | 0.335 | 0.440 | 0.005 | 0.675 | 1.030 | 1.023 | 0.988 | 1.115 | 0.0301 | 0.0231 | 0.0118 | 0.1151 |
| **U251** | 26.611 | 28.688 | 23.287 | 25.890 | 29.370 | 0.148 | 0.624 | 0.015 | 0.898 | 0.944 | 0.997 | 1.002 | 1.080 | 0.0559 | 0.0026 | 0.0018 | 0.0803 |
| **U87** | 27.356 | 25.261 | 19.813 | 22.705 | 27.559 | 0.861 | 0.204 | 0.005 | 0.346 | 0.971 | 0.878 | 0.852 | 0.947 | 0.0294 | 0.1217 | 0.1477 | 0.0526 |
| **SW780** | 31.012 | 30.075 | 23.475 | 25.409 | 28.393 | 6.146 | 3.235 | 0.033 | 1.264 | 1.100 | 1.046 | 1.010 | 1.060 | 0.1003 | 0.0456 | 0.0099 | 0.0602 |
| **HEPG2** | 32.706 | 30.537 | 24.964 | 26.895 | 28.075 | 24.788 | 5.512 | 0.116 | 4.415 | 1.160 | 1.062 | 1.074 | 1.122 | 0.1604 | 0.0617 | 0.0739 | 0.1222 |
| **OSC20** | 26.139 | 24.965 | 20.392 | 23.547 | 29.555 | 0.097 | 0.043 | 0.002 | 0.161 | 0.927 | 0.868 | 0.877 | 0.983 | 0.0726 | 0.1320 | 0.1227 | 0.0175 |
| **CAL27** | 25.802 | 27.120 | 22.171 | 23.766 | 29.569 | 0.077 | 0.191 | 0.006 | 0.188 | 0.915 | 0.943 | 0.954 | 0.992 | 0.0846 | 0.0571 | 0.0462 | 0.0084 |
| **CAL33** | 25.828 | 25.417 | 20.687 | 22.061 | 29.490 | 0.078 | 0.059 | 0.002 | 0.058 | 0.916 | 0.884 | 0.890 | 0.921 | 0.0836 | 0.1163 | 0.1101 | 0.0795 |
| **293T** | 27.572 | 29.716 | 22.191 | 23.607 | 28.977 | 0.406 | 1.767 | 0.010 | 0.258 | 0.978 | 1.033 | 0.955 | 0.985 | 0.0218 | 0.0332 | 0.0454 | 0.0150 |
| **H23S** | 29.708 | 31.033 | 26.302 | 27.336 | 27.649 | 4.198 | 10.493 | 0.394 | 8.052 | 1.054 | 1.079 | 1.131 | 1.141 | 0.0540 | 0.0790 | 0.1315 | 0.1406 |
| **H23R** | 31.031 | 32.730 | 27.914 | 27.196 | 26.735 | 19.869 | 63.817 | 2.265 | 13.785 | 1.101 | 1.138 | 1.201 | 1.135 | 0.1010 | 0.1380 | 0.2008 | 0.1348 |
| **H23R CBDCA** | 27.767 | 27.295 | 23.618 | 23.742 | 25.256 | 5.736 | 4.132 | 0.323 | 3.520 | 0.985 | 0.949 | 1.016 | 0.991 | 0.0149 | 0.0510 | 0.0160 | 0.0094 |
| **A2780S** | 28.749 | 29.710 | 25.847 | 23.775 | 26.148 | 6.079 | 11.827 | 0.817 | 1.934 | 1.020 | 1.033 | 1.112 | 0.992 | 0.0200 | 0.0329 | 0.1119 | 0.0079 |
| **A2780R** | 28.122 | 29.528 | 24.957 | 23.581 | 24.835 | 9.763 | 25.893 | 1.088 | 4.197 | 0.998 | 1.027 | 1.074 | 0.984 | 0.0023 | 0.0266 | 0.0736 | 0.0160 |
| **A2780R CBDCA** | 27.824 | 27.843 | 26.512 | 23.184 | 24.176 | 12.395 | 12.716 | 5.055 | 5.031 | 0.987 | 0.968 | 1.141 | 0.967 | 0.0128 | 0.0319 | 0.1405 | 0.0326 |
| **OVCARS** | 25.822 | 28.400 | 22.296 | 22.903 | 27.666 | 0.282 | 1.673 | 0.024 | 0.372 | 0.916 | 0.987 | 0.959 | 0.956 | 0.0838 | 0.0126 | 0.0409 | 0.0444 |
| **OVCAR R** | 28.500 | 32.031 | 24.059 | 25.754 | 28.591 | 0.946 | 10.948 | 0.043 | 1.404 | 1.011 | 1.114 | 1.035 | 1.075 | 0.0112 | 0.1137 | 0.0350 | 0.0746 |
| **41M** | 30.531 | 32.479 | 27.060 | 25.980 | 30.492 | 0.919 | 3.517 | 0.082 | 0.389 | 1.083 | 1.129 | 1.164 | 1.084 | 0.0832 | 0.1292 | 0.1641 | 0.0840 |
| **41MR** | 31.430 | 30.867 | 25.920 | 27.496 | 26.897 | 23.949 | 15.749 | 0.512 | 15.234 | 1.115 | 1.073 | 1.115 | 1.147 | 0.1151 | 0.0732 | 0.1150 | 0.1473 |
| **41M 2Gy** | 27.763 | 27.389 | 20.518 | 18.890 | 27.810 | 0.968 | 0.747 | 0.006 | 0.021 | 0.985 | 0.952 | 0.883 | 0.788 | 0.0150 | 0.0477 | 0.1173 | 0.2118 |
| **41M 4Gy** | 27.817 | 27.030 | 20.560 | 19.121 | 28.028 | 0.864 | 0.501 | 0.006 | 0.021 | 0.987 | 0.940 | 0.884 | 0.798 | 0.0131 | 0.0602 | 0.1155 | 0.2022 |
| **41M 6Gy** | 27.935 | 27.395 | 20.673 | 18.997 | 28.365 | 0.745 | 0.511 | 0.005 | 0.015 | 0.991 | 0.952 | 0.889 | 0.793 | 0.0089 | 0.0475 | 0.1107 | 0.2073 |

**Table S2.** Raw CT values of the endogenous miRNAs in sEVs compartment from 41M cells treated with radiotherapy at 0, 2, 4 and 6 Gy. Cycle number variation for each of the four miRNAs was analyzed using 41M mock cells without radiotherapy treatment as calibrator.

|  | **CT values** | | | | **Cycle number variation** | | | |
| --- | --- | --- | --- | --- | --- | --- | --- | --- |
|  | **miR-16-5p** | **miR-151a-3p** | **miR-221-3p** | **miR-22-5p** | **miR-16-5p** | **miR-151a-3p** | **miR-221-3p** | **miR-22-5p** |
| **41M** | 25.929 | 30.585 | 27.095 | 33.128 | 1 | 1 | 1 | 1 |
|  | 26.012 | 30.535 | 27.002 | 31.830 | 1 | 1 | 1 | 1 |
|  | 25.999 | 30.472 | 27.085 | 32.479 | 1 | 1 | 1 | 1 |
| **41M 2Gy** | 18.901 | 27.958 | 20.535 | 27.412 | 7.028 | 2.627 | 6.560 | 5.716 |
|  | 18.885 | 27.948 | 20.509 | 27.330 | 7.126 | 2.587 | 6.493 | 4.500 |
|  | 18.882 | 27.951 | 20.511 | 27.424 | 7.117 | 2.520 | 6.574 | 5.055 |
| **41M 4GY** | 19.139 | 27.853 | 20.561 | 26.944 | 6.790 | 2.732 | 6.534 | 6.184 |
|  | 19.094 | 27.901 | 20.578 | 27.070 | 6.918 | 2.635 | 6.423 | 4.760 |
|  | 19.130 | 27.822 | 20.540 | 27.075 | 6.869 | 2.650 | 6.545 | 5.404 |
| **41M 6GY** | 19.066 | 27.976 | 20.651 | 27.445 | 6.863 | 2.608 | 6.444 | 5.683 |
|  | 18.988 | 27.926 | 20.621 | 27.327 | 7.023 | 2.610 | 6.381 | 4.503 |
|  | 18.938 | 27.965 | 20.748 | 27.412 | 7.062 | 2.506 | 6.337 | 5.068 |

**Table S3:** Raw CT values of miR-151a-3p, miR-22-5p, miR-221-3p, miR-16-5p, miR-451a and its normalized levels using all the endogenous miRNAs in circulating sEVs from 14 NSCLC patients (nine advanced and five early stage), six ovarian cancer patients (three plasma and three paired ascitic fluid), five glioblastoma patients and five healthy volunteers. Normalized CT values are compared to the triplicate CT values obtained by qRT-PCR from each sample normalized against the mean value of all miRNAs analyzed in the total samples evaluated in the assay. Distance from the mean (DM) was calculated using the normalized values in terms of absolute values |1- Normalized CT values|

|  | **CT values** | | | | | **Normalized miR-451a values** | | | | **Normalized CT values** | | | | **DM** | | | |
| --- | --- | --- | --- | --- | --- | --- | --- | --- | --- | --- | --- | --- | --- | --- | --- | --- | --- |
|  | **miR-151a-3p** | **miR-22-5p** | **miR-221-3p** | **miR-16-5p** | **miR-451a** | **miR-151a-3p** | **miR-22-5p** | **miR-221-3p** | **miR-16-5p** | **miR 151a-3p** | **miR 22-5p** | **miR 221-3p** | **miR 16-5p** | **miR 151a-3p** | **miR 22-5p** | **miR 221-3p** | **miR 16-5p** |
| **A1** | 30.309 | 31.461 | 27.991 | 22.357 | 20.473 | 913.899 | 2031.038 | 183.358 | 369.108 | 1.012 | 1.019 | 1.045 | 0.936 | 0.012 | 0.019 | 0.045 | 0.064 |
| **A6** | 30.992 | 30.812 | 28.949 | 23.102 | 21.561 | 690.195 | 609.322 | 167.489 | 290.822 | 1.035 | 0.998 | 1.081 | 0.968 | 0.035 | 0.002 | 0.081 | 0.032 |
| **A13** | 29.708 | 29.773 | 26.694 | 21.978 | 19.927 | 879.918 | 920.335 | 108.952 | 414.335 | 0.992 | 0.964 | 0.997 | 0.920 | 0.008 | 0.036 | 0.003 | 0.080 |
| **A14** | 29.312 | 30.144 | 26.245 | 24.583 | 22.216 | 136.811 | 243.580 | 16.328 | 515.845 | 0.979 | 0.976 | 0.980 | 1.030 | 0.021 | 0.024 | 0.020 | 0.030 |
| **A18** | 30.975 | 31.944 | 27.281 | 25.166 | 22.901 | 269.519 | 527.301 | 20.813 | 480.741 | 1.034 | 1.034 | 1.019 | 1.054 | 0.034 | 0.034 | 0.019 | 0.054 |
| **A19** | 29.749 | 31.839 | 28.083 | 21.199 | 18.244 | 2906.400 | 12376.064 | 916.160 | 775.826 | 0.993 | 1.031 | 1.049 | 0.888 | 0.007 | 0.031 | 0.049 | 0.112 |
| **A21** | 30.023 | 32.923 | 27.905 | 23.920 | 21.458 | 378.809 | 2828.323 | 87.291 | 551.182 | 1.002 | 1.066 | 1.042 | 1.002 | 0.002 | 0.066 | 0.042 | 0.002 |
| **A22** | 30.623 | 31.913 | 27.724 | 25.877 | 23.412 | 148.167 | 362.093 | 19.858 | 552.179 | 1.022 | 1.033 | 1.035 | 1.084 | 0.022 | 0.033 | 0.035 | 0.084 |
| **A28** | 30.298 | 30.959 | 29.217 | 22.261 | 19.181 | 2219.913 | 3511.084 | 1049.743 | 845.563 | 1.012 | 1.003 | 1.091 | 0.932 | 0.012 | 0.003 | 0.091 | 0.068 |
| **L24** | 31.866 | 30.419 | 26.544 | 22.532 | 19.627 | 4833.697 | 1773.260 | 120.873 | 748.849 | 1.064 | 0.985 | 0.991 | 0.944 | 0.064 | 0.015 | 0.009 | 0.056 |
| **L26** | 30.362 | 30.919 | 26.414 | 24.080 | 21.351 | 516.062 | 759.079 | 33.426 | 662.902 | 1.014 | 1.001 | 0.986 | 1.009 | 0.014 | 0.001 | 0.014 | 0.009 |
| **L27** | 29.187 | 30.011 | 24.998 | 22.844 | 20.099 | 544.249 | 963.389 | 29.831 | 670.529 | 0.975 | 0.972 | 0.933 | 0.957 | 0.025 | 0.028 | 0.067 | 0.043 |
| **L28** | 30.002 | 30.532 | 26.394 | 25.660 | 24.436 | 47.382 | 68.409 | 3.887 | 233.749 | 1.002 | 0.989 | 0.986 | 1.075 | 0.002 | 0.011 | 0.014 | 0.075 |
| **L29** | 29.825 | 30.353 | 25.069 | 25.310 | 23.609 | 74.322 | 107.166 | 2.751 | 324.930 | 0.996 | 0.983 | 0.936 | 1.060 | 0.004 | 0.017 | 0.064 | 0.060 |
| **OV22 PL** | 29.810 | 30.549 | 25.653 | 22.870 | 20.779 | 523.238 | 873.461 | 29.328 | 426.088 | 0.995 | 0.989 | 0.958 | 0.958 | 0.005 | 0.011 | 0.042 | 0.042 |
| **OV23 PL** | 29.589 | 31.137 | 25.928 | 26.280 | 23.534 | 66.493 | 194.320 | 5.255 | 670.775 | 0.988 | 1.008 | 0.968 | 1.101 | 0.012 | 0.008 | 0.032 | 0.101 |
| **OV26 PL** | 30.981 | 30.570 | 26.752 | 24.071 | 22.500 | 357.237 | 268.735 | 19.052 | 297.113 | 1.034 | 0.990 | 0.999 | 1.008 | 0.034 | 0.010 | 0.001 | 0.008 |
| **OV22 LA** | 26.217 | 25.197 | 20.549 | 19.991 | 18.166 | 265.311 | 130.766 | 5.215 | 354.365 | 0.875 | 0.816 | 0.767 | 0.837 | 0.125 | 0.184 | 0.233 | 0.163 |
| **OV23 LA** | 27.185 | 27.249 | 23.983 | 22.187 | 21.700 | 44.780 | 46.807 | 4.867 | 140.121 | 0.908 | 0.882 | 0.896 | 0.929 | 0.092 | 0.118 | 0.104 | 0.071 |
| **OV26 LA** | 28.661 | 26.737 | 23.968 | 24.840 | 25.820 | 7.167 | 1.889 | 0.277 | 50.716 | 0.957 | 0.866 | 0.895 | 1.040 | 0.043 | 0.134 | 0.105 | 0.040 |
| **GB3** | 30.103 | 31.513 | 26.837 | 24.799 | 22.197 | 239.941 | 637.618 | 24.937 | 607.300 | 1.005 | 1.020 | 1.002 | 1.039 | 0.005 | 0.020 | 0.002 | 0.039 |
| **GB6** | 30.006 | 31.900 | 26.193 | 23.133 | 20.305 | 832.381 | 3092.404 | 59.214 | 710.028 | 1.002 | 1.033 | 0.978 | 0.969 | 0.002 | 0.033 | 0.022 | 0.031 |
| **GB8** | 31.083 | 31.694 | 25.709 | 24.407 | 22.093 | 508.417 | 776.519 | 12.259 | 497.199 | 1.038 | 1.026 | 0.960 | 1.022 | 0.038 | 0.026 | 0.040 | 0.022 |
| **GB9** | 31.142 | 34.024 | 25.869 | 24.999 | 22.284 | 464.059 | 3420.278 | 12.000 | 656.446 | 1.040 | 1.102 | 0.966 | 1.047 | 0.040 | 0.102 | 0.034 | 0.047 |
| **GB11** | 31.382 | 31.561 | 26.823 | 24.231 | 23.790 | 192.945 | 218.375 | 8.185 | 135.742 | 1.048 | 1.022 | 1.002 | 1.015 | 0.048 | 0.022 | 0.002 | 0.015 |
| **C1** | 30.864 | 34.354 | 29.423 | 26.601 | 22.578 | 312.015 | 3506.521 | 114.912 | 1624.859 | 1.031 | 1.112 | 1.099 | 1.114 | 0.031 | 0.112 | 0.099 | 0.114 |
| **C2** | 29.388 | 30.125 | 27.827 | 22.702 | 18.306 | 2167.659 | 3613.778 | 734.846 | 2105.490 | 0.981 | 0.976 | 1.039 | 0.951 | 0.019 | 0.024 | 0.039 | 0.049 |
| **C3** | 29.665 | 31.518 | 29.750 | 24.187 | 19.750 | 965.239 | 3486.015 | 1023.771 | 2166.171 | 0.991 | 1.021 | 1.111 | 1.013 | 0.009 | 0.021 | 0.111 | 0.013 |
| **C5** | 29.146 | 32.760 | 29.551 | 24.437 | 20.239 | 480.070 | 5876.758 | 635.545 | 1835.312 | 0.973 | 1.061 | 1.104 | 1.023 | 0.027 | 0.061 | 0.104 | 0.023 |
| **C6** | 30.034 | 31.557 | 29.044 | 25.698 | 22.253 | 219.985 | 632.162 | 110.722 | 1088.954 | 1.003 | 1.022 | 1.085 | 1.076 | 0.003 | 0.022 | 0.085 | 0.076 |

**Tables S4:** Raw CT values of miR-151a-3p, miR-22-5p, miR-221-3p, miR-16-5p, miR-451a and its normalized levels using all the endogenous miRNAs in circulating sEVs from 79 NSCLC patients (51 advanced and 28 early stage). Normalized CT values are compared to the triplicate CT values obtained by qRT-PCR from each sample normalized against the mean value of all miRNAs analyzed in the total samples evaluated in the assay. Distance from the mean (DM) was calculated using the normalized values in terms of absolute values |1- Normalized CT values|

| **Patientsa** | **CT values** | | | **Normalized miR-451a values** | | **DM** | |
| --- | --- | --- | --- | --- | --- | --- | --- |
|  | **miR-151a-3p** | **miR-16-5p** | **miR-451a** | **miR-151a-3p** | **miR-16-5p** | **miR-151a-3p** | **miR-16-5p** |
| **L1** | 30.766 | 24.647 | 24.637 | 913.899 | 369.108 | 0.0221 | 0.0669 |
| **L2** | 29.671 | 23.844 | 23.605 | 57.833 | 352.839 | 0.0213 | 0.0957 |
| **L3** | 29.893 | 24.677 | 23.697 | 578.331 | 385.639 | 0.0612 | 0.0118 |
| **L4** | 29.554 | 25.054 | 23.649 | 1258.502 | 325.267 | 0.0958 | 0.0025 |
| **L5** | 29.689 | 24.340 | 23.313 | 451.465 | 361.334 | 0.0556 | 0.0158 |
| **L6** | 28.640 | 22.903 | 21.273 | 690.195 | 290.822 | 0.0451 | 0.0358 |
| **L7** | 29.340 | 23.624 | 24.873 | 731.060 | 566.044 | 0.0182 | 0.0775 |
| **L8** | 29.216 | 23.799 | 18.487 | 2018.469 | 737.951 | 0.0494 | 0.1613 |
| **L9** | 30.101 | 24.537 | 22.333 | 1632.926 | 414.064 | 0.1206 | 0.0271 |
| **L10** | 31.809 | 27.956 | 25.384 | 215.721 | 250.428 | 0.0401 | 0.0190 |
| **L11** | 29.995 | 25.880 | 23.811 | 168.410 | 327.893 | 0.0915 | 0.1139 |
| **L12** | 30.842 | 25.505 | 24.593 | 438.714 | 556.531 | 0.0010 | 0.0265 |
| **L13** | 30.710 | 27.374 | 24.772 | 879.918 | 414.335 | 0.0018 | 0.0827 |
| **L14** | 30.234 | 25.036 | 24.451 | 136.811 | 515.845 | 0.0116 | 0.0261 |
| **L15** | 31.261 | 26.731 | 23.796 | 1074.349 | 432.778 | 0.0686 | 0.0094 |
| **L16** | 30.029 | 25.972 | 23.157 | 523.396 | 477.999 | 0.0708 | 0.0426 |
| **L17** | 30.470 | 26.074 | 23.914 | 296.797 | 432.534 | 0.0449 | 0.0387 |
| **L18** | 30.202 | 23.792 | 22.759 | 269.519 | 480.741 | 0.0445 | 0.0504 |
| **L19** | 29.705 | 22.558 | 20.717 | 2906.400 | 775.826 | 0.0032 | 0.1152 |
| **L20** | 29.858 | 24.096 | 20.329 | 327.600 | 756.957 | 0.0058 | 0.0180 |
| **L21** | 29.479 | 23.274 | 19.652 | 378.809 | 551.182 | 0.0124 | 0.0016 |
| **L22** | 31.973 | 26.440 | 24.550 | 148.167 | 552.179 | 0.0327 | 0.0801 |
| **L23** | 31.866 | 22.532 | 19.627 | 69.788 | 335.900 | 0.0708 | 0.0325 |
| **L24** | 30.362 | 24.080 | 21.351 | 1063.176 | 703.520 | 0.0842 | 0.0399 |
| **L25** | 29.187 | 22.844 | 20.099 | 1438.494 | 736.620 | 0.0832 | 0.0232 |
| **L26** | 30.002 | 25.660 | 24.436 | 4002.800 | 1088.372 | 0.0378 | 0.0712 |
| **L27** | 29.825 | 25.310 | 23.609 | 468.393 | 734.398 | 0.0281 | 0.0472 |
| **L28** | 33.679 | 27.777 | 25.430 | 2219.913 | 845.563 | 0.0217 | 0.0708 |
| **A1** | 30.309 | 22.357 | 20.473 | 1374.207 | 919.525 | 0.0361 | 0.1085 |
| **A2** | 30.286 | 26.252 | 24.433 | 513.307 | 1141.215 | 0.0601 | 0.0658 |
| **A3** | 31.470 | 24.241 | 22.294 | 2507.241 | 770.524 | 0.0684 | 0.1953 |
| **A4** | 32.495 | 23.899 | 22.198 | 595.183 | 887.447 | 0.0817 | 0.1166 |
| **A5** | 31.303 | 24.338 | 22.484 | 240.556 | 896.154 | 0.0384 | 0.0078 |
| **A6** | 30.992 | 23.102 | 21.561 | 396.412 | 697.720 | 0.0116 | 0.0198 |
| **A7** | 29.114 | 22.101 | 19.600 | 2114.979 | 1047.237 | 0.0113 | 0.0680 |
| **A8** | 28.190 | 20.094 | 17.211 | 1203.805 | 828.528 | 0.0453 | 0.0060 |
| **A9** | 33.230 | 24.607 | 22.557 | 1005.816 | 837.689 | 0.0001 | 0.0507 |
| **A10** | 30.843 | 24.415 | 23.090 | 361.286 | 904.658 | 0.0096 | 0.0039 |
| **A11** | 32.369 | 26.686 | 24.973 | 246.047 | 582.637 | 0.0249 | 0.0184 |
| **A12** | 29.624 | 23.324 | 20.847 | 1171.816 | 821.737 | 0.0166 | 0.0403 |
| **A13** | 29.708 | 21.978 | 19.927 | 897.480 | 1184.303 | 0.0240 | 0.0068 |
| **A14** | 29.312 | 24.583 | 22.216 | 475.189 | 970.269 | 0.0236 | 0.0258 |
| **A15** | 31.690 | 23.734 | 21.620 | 738.159 | 519.114 | 0.0152 | 0.0796 |
| **A16** | 31.755 | 24.980 | 22.723 | 284.212 | 512.998 | 0.0302 | 0.0414 |
| **A17** | 30.987 | 24.887 | 22.774 | 184.738 | 517.547 | 0.0034 | 0.0182 |
| **A18** | 30.975 | 25.166 | 22.901 | 1221.841 | 1196.916 | 0.0267 | 0.0077 |
| **A19** | 29.749 | 21.199 | 18.244 | 584.170 | 1166.370 | 0.0972 | 0.1224 |
| **A20** | 29.826 | 24.391 | 21.470 | 864.003 | 911.199 | 0.0379 | 0.0833 |
| **A21** | 30.023 | 23.920 | 21.458 | 1399.039 | 1387.982 | 0.0300 | 0.0772 |
| **A22** | 30.623 | 25.877 | 23.412 | 4613.445 | 1217.057 | 0.0796 | 0.2183 |
| **A23** | 27.556 | 23.179 | 21.431 | 394.258 | 807.873 | 0.0292 | 0.0324 |
| **A24** | 32.153 | 24.913 | 22.099 | 69.996 | 100.708 | 0.0375 | 0.0287 |
| **A25** | 32.123 | 24.513 | 21.632 | 66.983 | 118.025 | 0.0006 | 0.0048 |
| **A26** | 30.775 | 22.252 | 18.808 | 73.292 | 197.182 | 0.0080 | 0.0300 |
| **A27** | 28.822 | 22.827 | 19.950 | 59.923 | 264.738 | 0.0034 | 0.0457 |
| **A28** | 30.298 | 22.261 | 19.181 | 83.055 | 203.806 | 0.0011 | 0.0159 |
| **A29** | 28.584 | 21.360 | 20.735 | 165.077 | 309.681 | 0.0342 | 0.0440 |
| **A30** | 27.873 | 22.382 | 20.684 | 22.118 | 42.080 | 0.0106 | 0.0140 |
| **A31** | 27.626 | 19.280 | 20.632 | 1697.215 | 3973.328 | 0.0148 | 0.0066 |
| **A32** | 27.233 | 21.165 | 20.581 | 217.987 | 460.719 | 0.0150 | 0.0241 |
| **A33** | 28.517 | 23.771 | 20.529 | 85.934 | 594.977 | 0.0726 | 0.1669 |
| **A34** | 29.311 | 23.483 | 20.477 | 72.736 | 419.768 | 0.0115 | 0.0802 |
| **A35** | 29.988 | 22.330 | 18.942 | 76.046 | 188.148 | 0.0400 | 0.0645 |
| **A36** | 30.998 | 23.815 | 20.764 | 61.304 | 606.896 | 0.0356 | 0.1426 |
| **A37** | 29.652 | 22.744 | 19.678 | 55.039 | 149.933 | 0.0195 | 0.0450 |
| **A38** | 29.370 | 24.051 | 20.873 | 176.634 | 764.757 | 0.0542 | 0.1157 |
| **A39** | 28.918 | 23.517 | 20.975 | 117.155 | 703.978 | 0.0126 | 0.0840 |
| **A40** | 30.148 | 22.992 | 19.953 | 94.093 | 447.044 | 0.0275 | 0.0883 |
| **A41** | 30.366 | 24.122 | 20.556 | 173.963 | 204.567 | 0.0185 | 0.0069 |
| **A42** | 28.953 | 23.339 | 20.061 | 507.837 | 358.223 | 0.0017 | 0.0585 |
| **A43** | 29.204 | 22.052 | 19.676 | 738.549 | 1361.049 | 0.0068 | 0.0057 |
| **A44** | 28.759 | 22.967 | 20.608 | 908.425 | 1230.877 | 0.0059 | 0.0286 |
| **A45** | 29.552 | 24.395 | 22.023 | 171.615 | 370.832 | 0.0782 | 0.1036 |
| **A46** | 30.446 | 23.773 | 20.192 | 4833.697 | 748.849 | 0.0746 | 0.0596 |
| **A47** | 32.537 | 26.891 | 23.347 | 516.062 | 662.902 | 0.0239 | 0.0051 |
| **A48** | 28.530 | 21.963 | 18.775 | 544.249 | 670.529 | 0.0158 | 0.0465 |
| **A49** | 28.764 | 22.109 | 18.314 | 47.382 | 233.749 | 0.0117 | 0.0710 |
| **A50** | 27.294 | 18.727 | 15.122 | 74.322 | 324.930 | 0.0058 | 0.0564 |
| **A51** | 28.790 | 23.181 | 20.167 | 304.237 | 508.629 | 0.1357 | 0.1594 |

**Table S5:** Raw CT values of miR-151a-3p, miR-22-5p, miR-221-3p, miR-16-5p, miR-451a and its normalized levels using all the endogenous miRNAs in circulating sEVs from 35 ovarian cancer patients. Normalized CT values are compared to the triplicate CT values obtained by qRT-PCR from each sample normalized against the mean value of all miRNAs analyzed in the total samples evaluated in the assay. Distance from the mean (DM) was calculated using the normalized values in terms of absolute values |1- Normalized CT values|

|  | **PLASMA** | | | | | | | **ASCITIC FLUID** | | | | | | |
| --- | --- | --- | --- | --- | --- | --- | --- | --- | --- | --- | --- | --- | --- | --- |
| **Patients** | **CT values** | | | **Normalized miR-451a values** | | **DM** | | **CT values** | | | **Normalized miR-451a values** | | **DM** | |
|  | **miR-451a** | **miR-151a-3p** | **miR-16-5p** | **miR-151a-3p** | **miR-16-5p** | **miR-151a-3p** | **miR-16-5p** | **miR-451a** | **miR-151a-3p** | **miR-16-5p** | **miR-151a-3p** | **miR-16-5p** | **miR-151a-3p** | **miR-16-5p** |
| **OV1** | 21.811 | 29.892 | 23.468 | 270.749 | 315.403 | 0.00800 | 0.02046 | 23.422 | 27.418 | 24.913 | 15.954 | 281.169 | 0.07542 | 0.03986 |
| **OV2** | 18.745 | 29.300 | 21.942 | 1503.824 | 916.791 | 0.01197 | 0.08417 | 24.198 | 29.922 | 24.374 | 52.832 | 112.940 | 0.00901 | 0.01734 |
| **OV3** | 17.358 | 29.483 | 20.483 | 4466.658 | 872.512 | 0.00580 | 0.14507 | 22.208 | 29.610 | 22.607 | 169.137 | 131.778 | 0.00149 | 0.05643 |
| **OV4** | 25.583 | 32.240 | 26.346 | 100.926 | 169.783 | 0.08718 | 0.09967 | 29.351 | 28.305 | 24.903 | 0.484 | 4.581 | 0.04551 | 0.03942 |
| **OV5** | 22.712 | 32.543 | 24.034 | 910.440 | 249.926 | 0.09740 | 0.00315 | 23.325 | 33.012 | 24.877 | 823.828 | 293.082 | 0.11320 | 0.03833 |
| **OV6** | 21.044 | 31.341 | 23.669 | 1257.454 | 616.815 | 0.05685 | 0.01208 | 24.293 | 29.565 | 23.878 | 38.653 | 75.000 | 0.00302 | 0.00337 |
| **OV7** | 23.896 | 33.028 | 25.802 | 561.281 | 374.790 | 0.11376 | 0.07693 | 20.611 | 30.882 | 22.914 | 1235.167 | 493.516 | 0.04138 | 0.04359 |
| **OV8** | 24.187 | 31.552 | 26.537 | 164.954 | 510.154 | 0.06400 | 0.10765 | 20.548 | 27.334 | 22.207 | 110.344 | 315.791 | 0.07825 | 0.07309 |
| **OV9** | 16.143 | 27.229 | 27.304 | 2172.861 | 228912.430 | 0.08181 | 0.13963 | - | - | - | - | - | - | - |
| **OV10** | 24.942 | 33.511 | 25.161 | 380.001 | 116.440 | 0.13006 | 0.05020 | 23.977 | 31.539 | 20.549 | 188.951 | 9.295 | 0.06353 | 0.14229 |
| **OV11** | 23.018 | 28.210 | 24.700 | 36.567 | 321.042 | 0.04871 | 0.03097 | 28.414 | 27.205 | 25.856 | 0.433 | 16.988 | 0.08262 | 0.07921 |
| **OV12** | 22.816 | 29.970 | 25.899 | 142.357 | 847.083 | 0.01063 | 0.08100 | 22.289 | 28.394 | 24.789 | 68.811 | 565.563 | 0.04251 | 0.03467 |
| **OV13** | 24.480 | 27.537 | 23.978 | 8.327 | 70.626 | 0.07140 | 0.00081 | 33.146 | 26.976 | 23.765 | 0.014 | 0.150 | 0.09033 | 0.00807 |
| **OV14** | 18.404 | 30.675 | 24.985 | 4943.661 | 9576.488 | 0.03442 | 0.04286 | 25.947 | 29.443 | 26.518 | 11.285 | 148.547 | 0.00713 | 0.10682 |
| **OV15** | 23.979 | 31.836 | 29.132 | 231.821 | 3556.922 | 0.07356 | 0.21592 | 26.458 | 28.723 | 27.544 | 4.805 | 212.219 | 0.03143 | 0.14965 |
| **OV16** | 18.738 | 31.278 | 25.005 | 5953.712 | 7700.092 | 0.05474 | 0.04369 | 23.576 | 30.165 | 25.915 | 96.301 | 506.183 | 0.01721 | 0.08167 |
| **OV17** | 19.213 | 29.727 | 21.668 | 1463.051 | 548.609 | 0.00245 | 0.09558 | 29.324 | 28.211 | 25.369 | 0.462 | 6.445 | 0.04869 | 0.05886 |
| **OV18** | 20.180 | 28.751 | 22.573 | 380.398 | 525.555 | 0.03047 | 0.05781 | 24.137 | 27.616 | 23.023 | 11.148 | 46.198 | 0.06875 | 0.03904 |
| **OV19** | 19.357 | 29.960 | 22.255 | 1554.694 | 745.122 | 0.01028 | 0.07112 | 32.576 | 29.808 | 31.049 | 0.147 | 34.686 | 0.00516 | 0.29594 |
| **OV20** | 19.741 | 29.342 | 21.635 | 776.550 | 371.696 | 0.01054 | 0.09696 | 31.545 | 28.758 | 26.542 | 0.145 | 3.118 | 0.03023 | 0.10782 |
| **OV21** | 22.558 | 30.597 | 24.273 | 262.970 | 328.329 | 0.03177 | 0.01313 | 27.473 | 27.785 | 23.024 | 1.242 | 4.578 | 0.06305 | 0.03902 |
| **OV22** | 20.779 | 29.810 | 22.870 | 523.238 | 426.088 | 0.00525 | 0.04543 | 18.166 | 26.217 | 19.991 | 265.311 | 354.365 | 0.11591 | 0.16559 |
| **OV23** | 23.534 | 29.589 | 26.280 | 66.493 | 670.775 | 0.00220 | 0.09691 | 21.700 | 27.185 | 22.187 | 44.780 | 140.121 | 0.08329 | 0.07396 |
| **OV24** | 20.363 | 27.302 | 22.568 | 122.723 | 461.111 | 0.07934 | 0.05805 | - | - | - | - | - | - | - |
| **OV25** | 21.007 | 29.079 | 22.616 | 269.058 | 305.064 | 0.01942 | 0.05603 | 25.238 | 27.021 | 23.278 | 3.441 | 25.701 | 0.08882 | 0.02840 |
| **OV26** | 22.500 | 30.981 | 24.071 | 357.237 | 297.113 | 0.04472 | 0.00470 | 25.820 | 28.661 | 24.840 | 7.167 | 50.716 | 0.03350 | 0.03680 |
| **OV27** | 16.825 | 27.699 | 19.257 | 1876.832 | 539.570 | 0.06593 | 0.19623 | 28.407 | 25.186 | 21.653 | 0.107 | 0.927 | 0.15070 | 0.09621 |
| **OV28** | 21.427 | 29.447 | 23.323 | 259.477 | 372.225 | 0.00701 | 0.02651 | 21.668 | 26.794 | 21.788 | 34.925 | 108.661 | 0.09646 | 0.09060 |
| **OV29** | 20.815 | 29.811 | 22.543 | 510.600 | 331.064 | 0.00529 | 0.05910 | 28.265 | 26.273 | 24.210 | 0.251 | 6.015 | 0.11405 | 0.01051 |
| **OV30** | 19.364 | 25.923 | 20.621 | 94.304 | 238.935 | 0.12582 | 0.13931 | 23.203 | 25.806 | 23.048 | 6.074 | 89.790 | 0.12979 | 0.03801 |
| **OV31** | 26.408 | 30.604 | 27.046 | 18.332 | 155.637 | 0.03201 | 0.12887 | 34.401 | 28.578 | 24.989 | 0.018 | 0.147 | 0.03630 | 0.04302 |
| **OV32** | 21.237 | 29.606 | 23.366 | 330.654 | 437.357 | 0.00163 | 0.02473 | 23.508 | 28.355 | 22.724 | 28.766 | 58.079 | 0.04384 | 0.05151 |
| **OV33** | 18.131 | 29.648 | 21.133 | 2931.650 | 801.064 | 0.00022 | 0.11795 | 23.432 | 26.253 | 22.512 | 7.068 | 52.858 | 0.11471 | 0.06038 |
| **OV34** | 19.825 | 28.945 | 21.960 | 556.689 | 439.476 | 0.02392 | 0.08340 | 23.522 | 29.030 | 24.603 | 45.502 | 211.549 | 0.02107 | 0.02690 |
| **OV35** | 20.241 | 30.048 | 22.971 | 895.646 | 663.241 | 0.01327 | 0.04122 | 27.098 | 26.400 | 21.924 | 0.617 | 2.769 | 0.10974 | 0.08493 |

**Table S6:** Raw CT values of miR-151a-3p, miR-22-5p, miR-221-3p, miR-16-5p, miR-451a and its normalized levels using all the endogenous miRNAs in circulating sEVs from 12 glioblastoma patients and 13 healthy volunteers. Normalized CT values are compared to the triplicate CT values obtained by qRT-PCR from each sample normalized against the mean value of all miRNAs analyzed in the total samples evaluated in the assay. Distance from the mean (DM) was calculated using the normalized values in terms of absolute values |1- Normalized CT values|

| **Patients** | **CT values** | | | **Normalized miR-451a values** | | **DM** | |
| --- | --- | --- | --- | --- | --- | --- | --- |
|  | **miR-451a** | **miR-151a-3p** | **miR-16-5p** | **miR-151a-3p** | **miR-16-5p** | **miR-151a-3p** | **miR-16-5p** |
| **GB1** | 22.753 | 31.525 | 26.232 | 437.013 | 1115.076 | 0.06307 | 0.09491 |
| **GB2** | 25.721 | 32.118 | 26.490 | 84.291 | 170.413 | 0.08308 | 0.10567 |
| **GB3** | 22.197 | 30.103 | 24.799 | 239.941 | 607.300 | 0.01513 | 0.03510 |
| **GB4** | 24.059 | 30.134 | 25.672 | 67.389 | 305.854 | 0.01616 | 0.07153 |
| **GB5** | 20.660 | 28.137 | 23.216 | 178.118 | 588.134 | 0.05118 | 0.03097 |
| **GB6** | 20.305 | 30.006 | 23.133 | 832.381 | 710.028 | 0.01186 | 0.03445 |
| **GB7** | 25.360 | 33.635 | 28.377 | 309.641 | 809.158 | 0.13421 | 0.18441 |
| **GB8** | 22.093 | 31.083 | 24.407 | 508.417 | 497.199 | 0.04817 | 0.01873 |
| **GB9** | 22.284 | 31.142 | 24.999 | 464.059 | 656.446 | 0.05016 | 0.04342 |
| **GB10** | 23.492 | 32.018 | 26.172 | 368.426 | 640.814 | 0.07968 | 0.09240 |
| **GB11** | 23.790 | 31.382 | 24.231 | 192.945 | 135.742 | 0.05825 | 0.01137 |
| **GB12** | 25.501 | 31.997 | 25.992 | 90.295 | 140.530 | 0.07900 | 0.08487 |
| **C1** | 22.578 | 30.864 | 26.601 | 312.015 | 1624.859 | 0.04077 | 0.11028 |
| **C2** | 18.306 | 29.388 | 22.702 | 2167.659 | 2105.490 | 0.00899 | 0.05243 |
| **C3** | 19.750 | 29.665 | 24.187 | 965.239 | 2166.171 | 0.00035 | 0.00956 |
| **C4** | 19.051 | 29.080 | 23.248 | 1044.486 | 1833.752 | 0.01938 | 0.02965 |
| **C5** | 20.239 | 29.146 | 24.437 | 480.070 | 1835.312 | 0.01715 | 0.01997 |
| **C6** | 22.253 | 30.034 | 25.698 | 219.985 | 1088.954 | 0.01281 | 0.07261 |
| **C7** | 20.128 | 28.481 | 24.278 | 327.094 | 1775.263 | 0.03957 | 0.01332 |
| **C8** | 19.844 | 28.063 | 23.693 | 298.005 | 1440.814 | 0.05366 | 0.01108 |
| **C9** | 17.658 | 28.064 | 21.615 | 1356.983 | 1553.723 | 0.05364 | 0.09780 |
| **C10** | 19.213 | 27.611 | 23.254 | 337.373 | 1646.309 | 0.06891 | 0.02940 |
| **C11** | 19.796 | 29.147 | 22.936 | 653.259 | 881.752 | 0.01712 | 0.04268 |
| **C12** | 22.230 | 30.547 | 25.890 | 318.798 | 1264.170 | 0.03009 | 0.08064 |
| **C13** | 19.562 | 29.085 | 22.930 | 736.037 | 1032.677 | 0.01920 | 0.04293 |

**Table S7. Cell line authentication using GenePrintR10 kit (Promega, USA). Genomics Service of the iiBm CSIC-UAM.** This system allows simultaneous amplification and marking with different fluorochromes of the microsatellites D21S11, TH01, TPOX, vWA, Amelogenin, CSF1PO, D16S539, D7S820, D13S317 and D5S818. The profiles obtained were compared with those published for each cell line in the available databases, allowing for the identification and authentication of human cell lines, as well as the detection of intra-species contamination *(PMID: 7723277). **XY described for R Prieto and ATCC; XX described for A Ayuso, A Muñoz and A Cano. Misidentified. This cell line is not the original glioblastoma cell line established in 1968 at the University of Uppsala. As described in PubMed (PMID: 27582061) it is likely also a glioblastoma cell line but whose origin is unknown. See U-87MG Uppsala (CVCL_GP63) for the original U-87MG cell line.

| **Cell Line** | **Tumor Type** | **M musculus** | **D5S818** | **D13S317** | **D7S820** | **D16S539** | **VWA** | **TH01** | **AMEL** | **TPOX** | **CSF1PO** | **D21S11** | **Reference** | **Coincidence** |
| --- | --- | --- | --- | --- | --- | --- | --- | --- | --- | --- | --- | --- | --- | --- |
| REF: NCI-H23 |  | Negative | 12.13 | 12 | 9.10 | 11 | 16.17 | 6 | X | 8.9 | 10 | 30 | ATCC ® CRL-5800 |  |
| NCI-H23 | Lung | ******** | 12.13 | 12 | 9.10 | 11 | 16.17 | 6 | X | 8.9 | 10 | 30 |  | Yes |
| REF: NCI-H1299 |  | Negative | 11 | 12 | 10 | 12.13 | 16.17.18 | 6.9.3 | X | 8 | 12 | 32.2 | ATCC®CRL-5803 |  |
| NCI-H1299 | Lung | ******** | 11 | 12 | 10 | 12.13 | 16.18 | 6.9.3 | X | 8 | 12 | 32.2 |  | Yes |
| REF: NCI-H727 |  | Negative | 11.12 | 11 | 8.10 | 11.13 | 14.15 | 8 | X | 8 | 11.12 | 29.32.2 | ATCC ® CRL-5815 |  |
| NCI-H727 | Lung | ******** | 11.12 | 11 | 8.10 | 11.13 | 14.15 | 8 | X | 8 | 11.12 | 29.32.2 |  | Yes |
| REF: NCI-H1975 |  | Negative | 11.12 | 10.13 | 8.11 | 9.12 | 18 | 7 | X | 8.11 | 12 | 28.32.2 | ATCC ® CRL-5908/CVCL_1511 |  |
| NCI-H1975 | Lung | ******** | 11.12 | 10.13 | 8.11 | 9.12 | 18 | 7 | X | 8.11 | 12 | 28.32.2 |  | Yes |
| REF: A549 |  | Negative | 11 | 11 | 8.11 | 11.12 | 14 | 8.9.3 | X.Y | 8.11 | 10.12 | 29.00 | ATCC ® CCL-185 |  |
| A549 | Lung | ******** | 11 | 11 | 8.11 | 11.12 | 14 | 8.9.3 | X.Y | 8.11 | 10.12 | 29 |  | Yes |
| REF: HT-29 |  | Negative | 11.12 | 11.12 | 10 | 11.12 | 17.19 | 6.9 | X | 8.9 | 11.12 | 29.30 | ATCC ® HTB-38 |  |
| HT-29 | Colon | ******** | 11.12 | 11 | 10 | 11.12 | 17 | 6.9 | X | 8.9 | 11.12 | 29 |  | Yes |
| REF: LoVo |  | Negative | 11.12.13 | 8.11 | 9.3.10.11 | 9.12 | 17.18 | 9.3 | X.Y | 8.9 | 11.13.14 | 29.31.2 | ATCC® CCL-229 |  |
| LoVo | Colon | ******** | 11.13 | 8.11 | 10.11 | 9.12 | 17.18 | 9.3 | X.Y | 8.9 | 10.11.13.14 | 29.31.2 |  | Yes |
| REF: IMIM- PC2 |  | Negative | 12 | 11 | 8.1 | 11 | 14.16 | 7.9 | X | 11 | 11 | 29.30 | Dr. F. X. Real (*) |  |
| IMIM -PC2 | Pancreas | ******** | 12 | 11 | 8.1 | 11 | 14.16 | 7.9 | X | 11 | 11 | 29.30 |  |  |
| REF: PANC-1 |  | Negative | 11.13 | 11 | 8.10 | 11 | 15 | 7.8 | X | 8.11 | 10.12 | 28 | ATCC ® CRL-1469 |  |
| PANC-1 | Pancreas | ******** | 11.13 | 11 | 8.10 | 11 | 15 | 7.8 | X | 8.11 | 10.12 | 28 |  | Yes |
| REF: MCF7 |  | Negative | 11.12 | 11 | 8.9 | 11.12 | 14.15 | 6 | X | 9.12 | 10 | 30 | ATCC ® HTB-22 |  |
| MCF | Breast | ******** | 11.12 | 11 | 8.9 | 11.12 | 14.15 | 6 | X | 9.12 | 10 | 30 |  | Yes |
| REF: T98G |  | Negative | 10.12 | 13 | 9.10 | 13 | 17.20 | 7.9.3 | X.Y | 8 | 10.12 | 28.32.2 | ATCC®CRL-1690 |  |
| T98G | Brain | ******** | 10.12 | 13 | 9.10 | 13 | 17.20 | 7.9.3 | X.Y | 8 | 10.12 | 28.32.2 |  | Yes |
| REF: LN-229 |  | Negative | 11.12 | 10.11 | 8.11 | 12 | 16.19 | 9.3 | X | 8 | 12.00 | 29.30 | ATCC®CRL-2611 |  |
| LN-229 | Brain | ******** | 11.12 | 10.11 | 8.11 | 12 | 16.19 | 9.3 | X | 8 | 12.00 | 29.3 |  | Yes |
| REF: U-251MG |  | Negative | 11.12 | 10.11 | 10.12 | 12 | 16.18 | 9.3 | X | 8 | 11.12 | 29. 30 | SIGMA/CVCL_0021 |  |
| U-251MG | Brain | ******** | 11.12 | 10.11 | 10.12 | 12 | 16.18 | 9.3 | X | 8 | 11.12 | 29. 30 |  | Yes |
| REF: U-87MG |  | Negative | 11.12 | 8.11 | 8.9 | 12 | 15.17 | 9.3 | X.(Y) | 8 | 10.11 | 28.32.2 | ATCC ® HTB-14 / CVCL_0022 (**) |  |
| U-87MG | Brain | ******** | 11.12 | 8.11 | 8.9 | 12 | 15.17 | 9.3 | X.(Y) | 8 | 10.11 | 28.32.2 |  | Yes |
| REF: SW 780 |  | Negative | 11.12 | 11.12 | 9.10 | 9.11 | 16.19 | 6 | X | 8 | 10.11 | 29.30 | ATCC ® CRL-2169 |  |
| SW 780 | Bladder | ******** | 11.12 | 11.12 | 9.10 | 9.11 | 16.19 | 6 | X | 8 | 10.11 | 29.30 |  | Yes |
| REF: Hep G2 |  | Negative | 11.12 | 9.13 | 10 | 12.13 | 17 | 9 | X.Y | 8.9 | 10.11 | 29.31 | ATCC ® HB-8065 |  |
| Hep G2 | Liver | ******** | 11.12 | 9.13 | 10 | 12.13 | 17 | 9 | X.Y | 8.9 | 10.11 | 29.31 |  | Yes |
| REF: CAL 27 |  | Negative | 11.12 | 10.11 | 10.00 | 11.12 | 14.17 | 6.9.3 | X | 8 | 10.12 | 28.29 | ATCC ® CRL-2095/CVCL_1107 |  |
| CAL 27 | Head and neck | ******** | 11.12 | 10.11 | 10.00 | 11.12 | 14.17 | 6.9.3 | X | 8 | 10.12 | 28.29 |  | Yes |
| REF: CAL-33 |  | Negative | 11.12 | 8.13 | 8.10 | 11 | 17 | 9.9.3 | X.Y | 8 | 11.12 | 29.30 | CVCL_1108 |  |
| CAL-33 | Head and neck | ******** | 11.12 | 8.13 | 8.10 | 11 | 17 | 9.9.3 | X.Y | 8 | 11.12 | 29.30 |  | Yes |
| REF: OSC-20 |  | Negative | 13 | 8 | 10.00 | 9.13 | 18 | 9.9.3 | X | 8.11 | 12.13 | 30.00 | CVCL_3087 |  |
| OCS-20 | Head and neck | ******** | 13 | 8 | 10.00 | 9.13 | 18 | 9.9.3 | X | 8.11 | 12.13 | 30.00 |  | Yes |
| REF: 293T |  | Negative | 8.9 | 12.14 | 11.00 | 9.13 | 16.19 | 7.9.3 | X | 11 | 11.12 | 28.30.2 | ATCC®CRL-3216 |  |
| 293T | Embryo kidney | ******** | 8.9 | 12.14 | 11.00 | 9.13 | 16.19 | 7.9.3 | X | 11 | 11.12 | 28.30.2 |  | Yes |
| REF: A2780 |  | Negative | 11.12 | 12.13 | 10 | 11.13 | 15.16 | 6 | X | 8.10 | 10.11 | 28.00 | CVCL_0134 |  |
| A2780 | Ovary | ******** | 11.12 | 12.13 | 10 | 11.12.13 | 15.16 | 6 | X | 8.10 | 10.11 | 28 |  | Yes |
| REF: OVCAR-3 |  | Negative | 11.12 | 12 | 10 | 12 | 17 | 9.9.3 | X | 8.00 | 11.12 | 29.31.2 | ATCC ® HTB-161 |  |
| OVCAR-3 | Ovary | ******** | 11.12 | 12 | 10 | 12 | 17 | 9.9.3 | X | 8.00 | 11.12 | 29.31.2 |  | Yes |
